# Profiling of HIV-1 elite neutralizer cohort reveals a CD4bs bnAb for HIV-1 prevention and therapy

**DOI:** 10.1038/s41590-025-02286-5

**Published:** 2025-10-06

**Authors:** Lutz Gieselmann, Andrew T. DeLaitsch, Malena Rohde, Henning Gruell, Christoph Kreer, Meryem Seda Ercanoglu, Harry B. Gristick, Philipp Schommers, Elvin Ahmadov, Caelan Radford, Andrea Mazzolini, Lily Zhang, Anthony P. West, Johanna Worczinski, Anna Ashurov, Maren L. Reichwein, Jacqueline Knüfer, Ricarda Stumpf, Nonhlanhla N. Mkhize, Haajira Kaldine, Sinethemba Bhebhe, Sharvari Deshpande, Federico Giovannoni, Erin Stefanutti, Fabio Benigni, Colin Havenar-Daughton, Davide Corti, Arne Kroidl, Anurag Adhikari, Aubin J. Nanfack, Georgia E. Ambada, Ralf Duerr, Lucas Maganga, Wiston William, Nyanda E. Ntinginya, Timo Wolf, Christof Geldmacher, Michael Hoelscher, Clara Lehmann, Penny L. Moore, Thierry Mora, Aleksandra M. Walczak, Peter B. Gilbert, Nicole A. Doria-Rose, Yunda Huang, Jesse D. Bloom, Michael S. Seaman, Pamela J. Bjorkman, Florian Klein

**Affiliations:** 1 Laboratory of Experimental Immunology, Institute of Virology, Faculty of Medicine and University Hospital Cologne, University of Cologne, Cologne, Germany; 2 German Center for Infection Research, Partner Site Bonn-Cologne, Cologne, Germany; 3 Divison of Biology and Biological Engineering, California Institute of Technology, Pasadena, CA USA; 4 Center for Molecular Medicine Cologne (CMMC), University of Cologne, Cologne, Germany; 5 Department I of Internal Medicine, Faculty of Medicine and University Hospital Cologne, University of Cologne, Cologne, Germany; 6 Basic Sciences Division, Fred Hutch Cancer Center, Seattle, WA USA; 7 Laboratoire de Physique de l’Ecole Normale Supérieure, CNRS, Paris Sciences and Lettres University, Sorbonne Université and Université Paris-Cité, Paris, France; 8 Vaccine and Infectious Disease Division, Fred Hutchinson Cancer Center, Seattle, WA USA; 9 SAMRC Antibody Immunity Research Unit, Faculty of Health Sciences, University of the Witwatersrand, Johannesburg, South Africa; 10 Center for HIV and STIs, National Institute for Communicable Diseases a Division of the National Health Laboratory Service, Johannesburg, South Africa; 11 Vir Biotechnology, San Francisco, CA USA; 12 Institute of Infectious Diseases and Tropical Medicine, LMU University Hospital, Munich, Germany; 13 German Center for Infection Research (DZIF), Partner Site, Munich, Germany; 14 Department of Infection and Immunology, Kathmandu Research Institute for Biological Sciences, Lalitpur, Nepal; 15 Chantal BIYA International Reference Centre for Research on HIV/AIDS Prevention and Management, Yaoundé, Cameroon; 16 Department of Microbiology, New York University School of Medicine, New York City, NY USA; 17 Department of Medicine, NYU Grossman School of Medicine, New York City, NY USA; 18 Vaccine Center, NYU Grossman School of Medicine, New York City, NY USA; 19 Mbeya Medical Research Centre, National Institute for Medical Research, Mbeya, Tanzania; 20 Department for Infectious Diseases, Centre of Internal Medicine II, University Hospital Frankfurt, Frankfurt am Main, Germany; 21 Fraunhofer Institute for Translational Medicine and Pharmacology ITMP, Immunology, Infection and Pandemic Research, Munich, Germany; 22 Unit Global Health, Helmholtz Zentrum München, German Research Center for Environmental Health (HMGU), Neuherberg, Germany; 23 Center for the AIDS Programme of Research in South Africa (CAPRISA), Durban, South Africa; 24 Department of Biostatistics, University of Washington, Seattle, WA USA; 25 Vaccine Research Center, National Institute of Allergy and Infectious Diseases, National Institutes of Health, Bethesda, MD USA; 26 Department of Global Health, University of Washington, Seattle, WA USA; 27 Basic Sciences and Computational Biology, Fred Hutchinson Cancer Research Center, Seattle, WA USA; 28 Howard Hughes Medical Institute, Chevy Chase, MD USA; 29 Center for Virology and Vaccine Research, Beth Israel Deaconess Medical Center, Harvard Medical School, Boston, MA USA

**Keywords:** HIV infections, Humoral immunity

## Abstract

Administration of HIV-1 neutralizing antibodies can suppress viremia and prevent infection in vivo. However, clinical use is challenged by envelope diversity and rapid viral escape. Here, we performed single B cell profiling of 32 top HIV-1 elite neutralizers to identify broadly neutralizing antibodies with highest antiviral activity. From 831 expressed monoclonal antibodies, we identified 04_A06, a V_H_1-2-encoded broadly neutralizing antibody to the CD4 binding site with remarkable breadth and potency against multiclade pseudovirus panels (geometric mean half-maximal inhibitory concentration = 0.059 µg ml^−1^, breadth = 98.5%, 332 strains). Moreover, 04_A06 was not susceptible to classic CD4 binding site escape variants and maintained full viral suppression in HIV-1-infected humanized mice. Structural analyses revealed an unusually long 11-amino-acid heavy chain insertion that facilitates interprotomer contacts with highly conserved residues on the adjacent gp120 protomer. Finally, 04_A06 demonstrated high activity against contemporaneously circulating viruses from the Antibody-Mediated Prevention trials (geometric mean half-maximal inhibitory concentration = 0.082 µg ml^−1^, breadth = 98.4%, 191 virus strains), and in silico modeling for 04_A06LS predicted prevention efficacy of >93%. Thus, 04_A06 will provide unique opportunities for effective treatment and prevention of HIV-1 infection.

## Abstract (web summary)

Klein and colleagues characterize 04_A06, a new V_H_1-2-encoded broadly neutralizing antibody that has marked breadth and potency against extended multiclade HIV-1 pseudovirus panels and can maintain full viral suppression in HIV-1-infected humanized mice.

## Main

Broadly neutralizing antibodies (bnAbs), capable of neutralizing diverse HIV-1 strains and subtypes, represent promising tools for immunotherapy and prevention^1^. bnAbs target conserved epitopes on the HIV-1 Env trimer, including the CD4 binding site (CD4bs), glycan-dependent sites at the V3 base and V2 region, the gp120–gp41 interface, the membrane-proximal external region, the fusion peptide and the silent face^2^. The highly conserved CD4bs is critical for virus engagement of host cells. Given its pivotal role in the viral lifecycle, many bnAbs to the CD4bs display high levels of antiviral activity, and viral evasion may entail substantial fitness costs^3^. Therefore, bnAbs to the CD4bs are prime targets for clinical evaluation and vaccine development^4^.

bnAbs to the CD4bs are categorized by genetic and structural features into VRC01-class (for example, VRC01, 3BNC117, N6, N49P7 and VRC07_523-LS_)^5–9^ and non-VRC01-class (for example, CH103, 8ANC131 and 1-18)^10,11^. VRC01-class members, encoded by the immunoglobulin heavy chain gene segment *IGHV1-2*, include a five-residue light chain complementary-determining region 3 (CDRL3) and are characterized by high somatic hypermutation^8,12^. Although bnAbs can reduce viremia, delay viral rebound and prevent infection with sensitive viruses, clinical trials have highlighted limitations, such as HIV-1 Env diversity and pre-existing and de novo resistance^1,13^, impeding clinical applicability. Therefore, identification of bnAbs with enhanced potency and breadth and restricted viral escape pathways remains critical.

Most bnAbs in clinical testing were isolated from a few HIV-1 elite neutralizers^6,7,9–11^. However, as both elite neutralizers and bnAb lineages within these individuals are rare, using large-scale screening can support discovery of new bnAbs with promising clinical potential. Here, we combined microscale antibody production with direct functional testing^14^ to perform detailed single-cell profiling of the largest cohort of top HIV-1 elite neutralizers studied to date (32 individuals). We identified 04_A06, a highly broad and potent bnAb to the CD4bs, from one of three genetically divergent B cell clones with overlapping CD4bs specificity. 04_A06 contains an 11-amino-acid insertion in the framework region heavy chain 1 (FWRH1) that contacts highly conserved Env residues (>99%), providing a structural explanation for its remarkable antiviral activity. This insertion also allowed 04_A06 to overcome classic viral CD4bs escape and to achieve full suppression of viremia in HIV-1_YU2_-infected humanized mice. Finally, based on high neutralizing activity of 04_A06 against transmitted viruses from two Antibody-Mediated Prevention (AMP) trials^13^, modeling predicted a 93% prevention efficacy (PE) for an extended half-life variant, supporting 04_A06 as a promising candidate for treatment and prevention.

## Results

### Large-scale profiling of HIV-1 neutralizers reveals new bnAbs

**Fig. 1:**
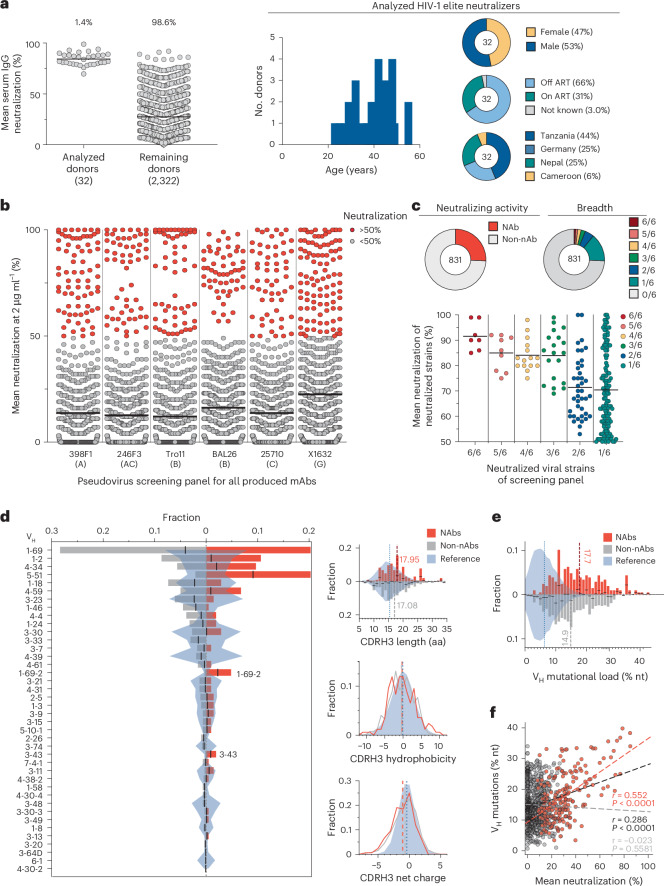
Large-scale isolation of mAbs from HIV-1 elite neutralizers. **a**, Dot plots illustrating the neutralizing activity of purified serum IgG samples from each donor (*n* = 2,354) against an HIV-1 global pseudovirus panel^15^ (left) and pie charts showing the distribution of age, sex, ART status and geographical origin of the 32 HIV-1 elite neutralizers^16^ (right). Serum IgG samples were tested in duplicate^16^; NA, not available. **b**, Neutralizing activity of 831 isolated mAbs against a screening pseudovirus panel of six viral strains from different clades (A, AC, B, C, G). Each antibody was evaluated at a concentration of 2 μg ml^−1^. Antibodies that achieved greater than 50% neutralization were classified as neutralizing (red). Antibodies with <50% neutralization were categorized as non-neutralizing mAbs (gray). Antibodies were tested once. **c**, Pie charts showing the proportion of nAbs and non-nAbs among the 831 isolated mAbs (top left), breadth (top right) and neutralization spectrum (bottom) of nAbs across HIV-1 strains of the screening panel. **d**, V_H_ gene segment usage (left) and CDRH3 characteristics (right) of 831 nAbs and non-nAbs, with V gene segments ordered by the overall frequencies in all 831 antibodies and bar graphs showing fractions of nAbs and non-nAbs among 831 mAbs calculated for individual subsets. CDRH3 characteristics comprise lengths in amino acids, cumulative hydrophobicities (Eisenberg scale) and net charge. Next-generation sequencing reference data from 48 healthy individuals^17^ are shown (blue); aa, amino acids. **e**, Bar graphs illustrating V_H_ mutational load of 831 non-nAbs and nAbs; nt, nucleotides. **f**, Correlation between V_H_ mutations and neutralizing activity of antibodies. Pearson correlation coefficients (*r*) and two-sided *P* values for nAbs (red, *P* < 0.0001), non-nAbs (gray, *P* = 0.5581) and all (black, *P* < 0.0001) mAbs are reported. Dashed lines show linear regressions for the respective subset. Center lines in dot plots in **a**–**c** indicate means. Black lines within bar graphs in **d** and **e** depict the center of combined fractions to indicate overrepresentation in either nAbs or non-nAbs. Dashed lines represent distribution means.

**Extended Data Fig. 1:**
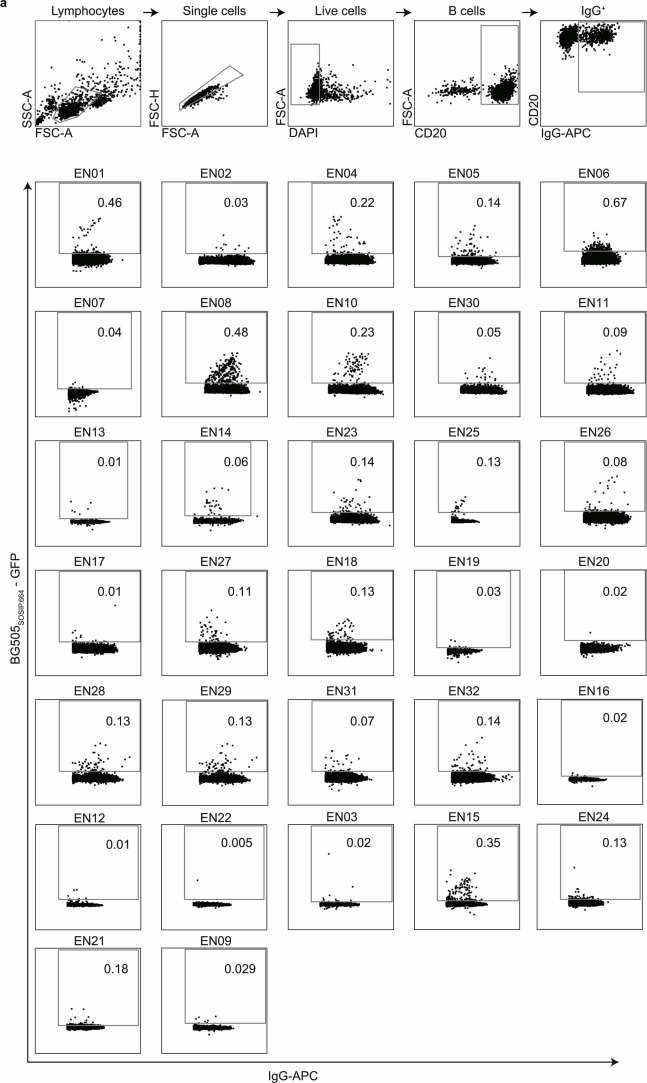
Gating strategy and single cell sorts of HIV-1-reactive B cell subsets. **a**, Individual FACS plots depicting sorting gates and frequencies of HIV-1-reactive, IgG^+^ B cells (in %) isolated from 32 donors. Numbers indicate the frequency of HIV-1 Env-reactive IgG+ B cells from the parental gate.

**Extended Data Fig. 2:**
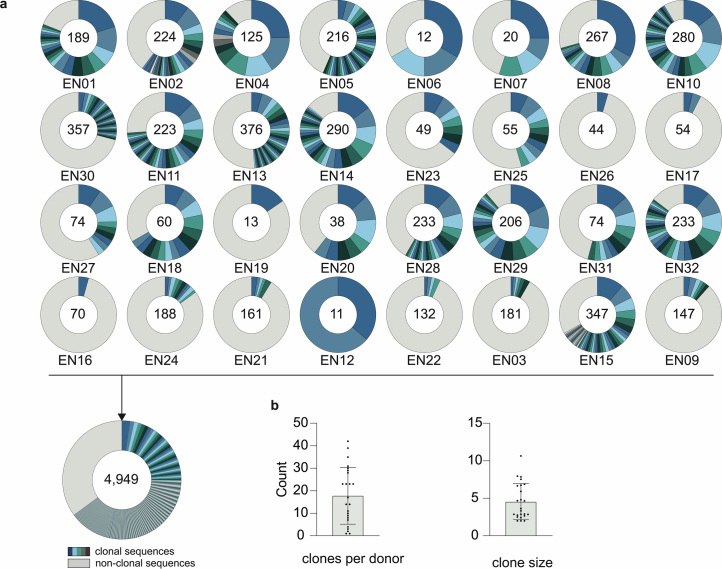
Analysis of isolated heavy chain sequences. **a**, Clonal relationship of heavy chain sequences amplified from single HIV-1-reactive IgG+ B cells isolated from 32 donors. Individual clones are colored in shades of blue, gray, and white. In the center of each pie chart, numbers of productive heavy chain sequences are illustrated. Presentation of clone sizes are proportional to the total number of productive heavy chain sequences per clone. **b**, Dot plot bar graphs showing the mean ± SD of the number of clones per donor (left) and clone size (right) across 32 HIV-1 elite neutralizers, with individual donor values indicated by dots.

In an international cohort of 2,354 people living with HIV-1 (PLWH), we ranked individuals by serum IgG neutralizing activity against the 12-strain HIV-1 global pseudovirus panel^15,16^. From 32 identified elite neutralizers, we collected blood samples to isolate new bnAbs. Donors were aged 21–53 years (median of 38 years), 47% female and 66% off antiretroviral therapy (ART) at the time of blood draw (Fig. 1a and Supplementary Table 1). Samples were obtained in Tanzania (44%), Germany (25%), Nepal (25%) and Cameroon (6%; Fig. 1a and Supplementary Table 1). HIV-1 Env-reactive DAPI^−^CD20^+^IgG^+^ memory B cells were isolated by single-cell sorting using green fluorescent protein (GFP)-labeled BG505_SOSIP.664_ and YU2_gp140_ baits, yielding frequencies of 0.005–0.67% (median 0.1%; Extended Data Fig. 1a). From 5,324 isolated cells, 4,949 IgG heavy chains and 2,256 light chains were amplified (2,255 heavy and light chain pairs) applying optimized PCR protocols^14^. In each individual, 4.3–100% of sequences were clonally related with a mean of 16 clones per individual and a mean clone size of 4 (Extended Data Fig. 2a,b). To identify neutralizing antibodies (nAbs), we expressed 831 mAbs from 27 donors and tested each for neutralizing activity against a screening panel of six HIV-1 pseudoviruses representative of different clades (Fig. 1b,c and Supplementary Table 2). Antibodies were selected to represent all identified B cell clones, and additionally, nonclonal mAbs that exhibited V_H_ gene sequences with uncommon features, such as high levels of somatic hypermutation (≤80% V_H_ gene germline identity), amino acid insertions or deletions and long CDRH3 regions (≥25 amino acids in length), were included. At a concentration of 2 µg ml^−1^, 214 (25%) mAbs displayed ≥50% neutralization against at least one virus strain (Fig. 1b,c and Supplementary Table 2). Most nAbs demonstrated activity against a single strain (124/214, 58%), and only seven mAbs from two donors (3.3%) neutralized all strains of the screening panel (Fig. 1c). Enhanced potency of nAbs was associated with breadth (Fig. 1c). Compared to healthy reference memory B cell repertoires^17^, the mAbs isolated from the 32 HIV-1 elite neutralizers demonstrated enriched V_H_ gene segments (for example, V_H_1-69, V_H_1-2 and V_H_4-34), longer CDRH3s and higher V_H_ mutation frequencies (Fig. 1d). Among nAbs, we detected higher fractions of V_H_5-51, V_H_1-69–2 and V_H_3-43 gene segments than in non-nAbs (Fig. 1d). On average, nAbs displayed longer CDRH3s and reduced net charge but no differences in hydrophobicity compared to non-nAbs (Fig. 1d). In addition, nAbs acquired more V_H_ gene mutations than non-nAbs, which correlated with higher neutralizing activity (Fig. 1e,f). We concluded that HIV-1 nAbs emerged from diverse V genes with preference for V_H_5-51, V_H_1-69-2 and V_H_3-43 compared to non-nAbs and are characterized by a high degree of somatic mutations correlating with antiviral activity^17^.

### Phylogenetic analyses reveal coexistence of distinct bnAbs to the CD4bs

**Extended Data Fig. 3:**
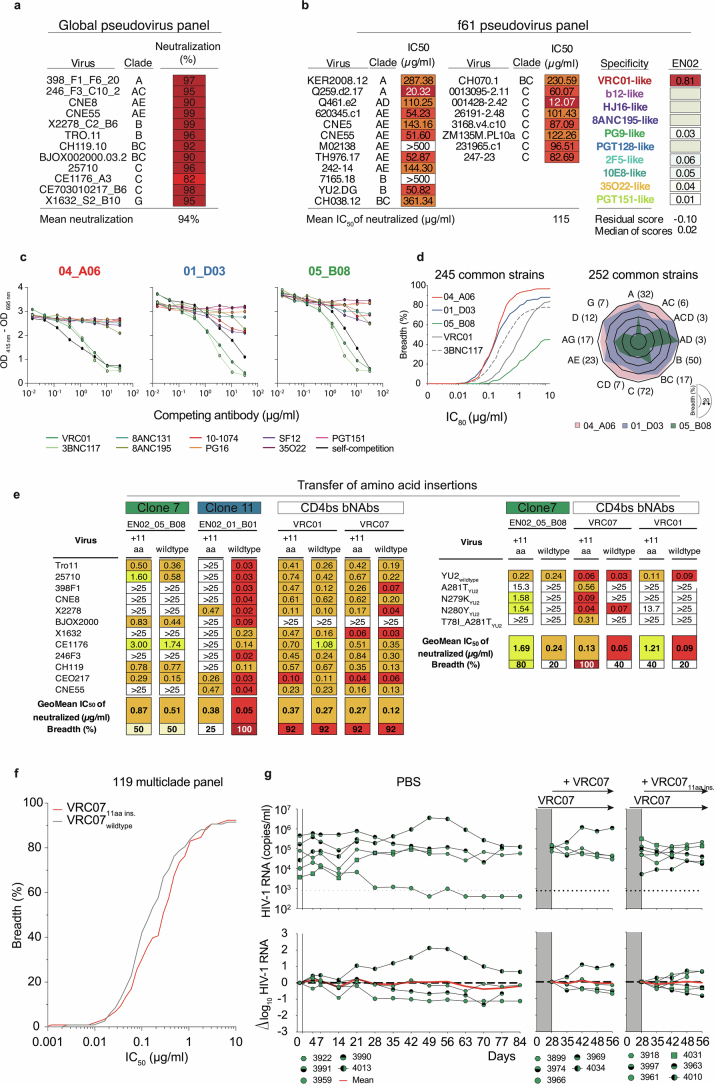
Neutralization and binding profile of serum, isolated and chimeric mAbs from donor EN02. **a**, Neutralizing activity of EN02 serum IgG against the HIV-1 global and **b**, f61 pseudovirus panel retrieved from^16^. Right panels show delineation scores of f61 panel-based computational epitope mapping. **c**, Interference of indicated bNAbs with reference bNAbs targeting known epitopes on the HIV-1 Env trimer measured by competition ELISAs. **d**, Neutralization breadth (%) and potency (GeoMean IC_80_) of representative bNAb 04_A06 (clone 9; red), 01_D03 (clone 11, blue); and 05_B08 (clone 7, green) against ≥245 pseudovirus strains. Samples were tested in duplicates. Neutralization data for reference bNAbs was retrieved from CATNAP database^49^. **e**, The elongated heavy chain FWRH1 of 04_A06 was engrafted into clonally distinct bNAbs isolated from donor EN02 and CD4bs reference bNAbs. The antiviral activity of wildtype and chimeric versions of isolated and reference mAbs was determined against the global HIV-1 panel and common CD4bs escape pseudovirus variants. **f**, Breadth (%) and potency (IC_50_) of the chimeric VRC07 antibody version against the 119 multiclade panel. **g**, Sequential treatment with the chimeric VRC07 antibody version (VRC07_11aa ins_) in HIV-1_YU2_-infected humanized mice following viral rebound during VRC07 monotherapy. This approach included maintaining VRC07 monotherapy while integrating VRC07_11aa ins._ in the treatment regimen. Mice treated with a double dose of VRC07 (1 mg) were included as a control group. Dashed lines in the top graphs indicate the lower limit of quantitation of the qPCR assay (LLQ) (784 copies/ml). Red lines display the average log_10_ changes compared to baseline viral loads (day 0).

Among all screened mAbs, those derived from individual EN02, a female living with HIV-1 clade C in Tanzania, exhibited the highest levels of breadth (up to 100%) and mean neutralization potency (up to 99%) against the HIV-1 pseudovirus screening panel^16^ (Supplementary Table 2). Purified EN02 serum IgG displayed broad and potent activity with a coverage of 100% and a mean serum IgG neutralization of 94% against the 12-strain global HIV-1 pseudovirus panel^15,16^ (Extended Data Fig. 3a and Supplementary Table 1). Based on serum IgG activity, EN02 was ranked as the second top elite neutralizer within the cohort of 2,354 PLWH^16^. Neutralization fingerprinting of purified serum IgG revealed VRC01-like activity (Extended Data Fig. 3b), suggesting neutralization to be predominantly mediated by antibodies to the CD4bs^16^.

**Fig. 2:**
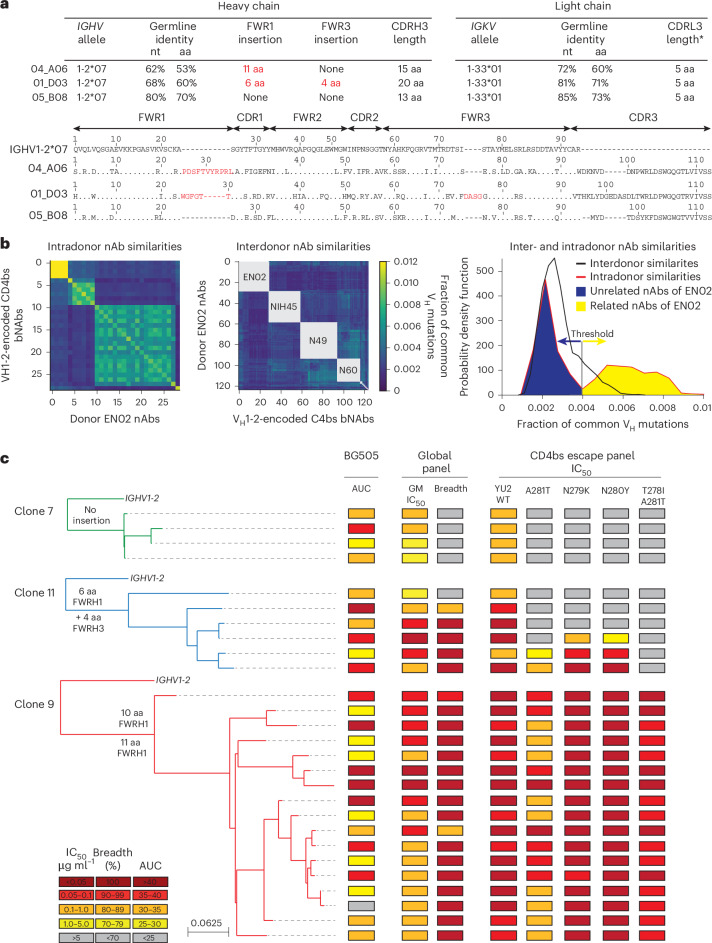
bnAbs coevolved with overlapping specificity for the CD4bs. **a**, Antibody characteristics and heavy chain sequence alignment of 04_A06, 01_D03 and 05_B08 to the germline *IGHV1-2* gene. Asterisk indicates that CDRL3 could not be determined using IMGT software, as the sequence did not fulfill criteria of the numbering scheme and CDR3 sequence definition. The stated CDRL3 is defined on the assumption of an amino acid substitution in the J-region motif (F/W-G-X-G). **b**, Heat maps illustrating the V_H_ sequence similarities of isolated antibodies within (intradonor, left) and between (interdonor, middle) donors. Donors for the comparator V_H_1-2-encoded CD4bs bnAbs are indicated in the center of each gray square. The corresponding histogram outlinines intra- and interdonor similarity distributions (right). The red line indicates the intradonor similarity distribution for donor EN02, the black line indicates the interdonor similarity distribution between nAbs from donor EN02 and V_H_1-2-encoded comparator bnAbs isolated from other donors. The gray line indicates the threshold for identification of clonally related sequences. Shaded areas under the intradonor similarity distribution graph indicate nonclonal (blue) and clonal (yellow) sequences. **c**, Maximum-likelihood phylogenetic trees of isolated bnAbs encoded by *IGHV1-2* originating from B cell clone 7, B cell clone 11 or B cell clone 9 and heat maps illustrating binding (mean area under the curve (AUC)) to BG505_SOSIP.664_ and neutralizing activity (GeoMean (GM) IC_50_ and breadth) against an HIV-1 global pseudovirus panel^15^ and common CD4bs escape variants. Samples were tested in duplicate. WT, wild-type.

Most nAbs isolated from donor EN02 were encoded by V_H_1-2*07 paired with V_K_1-33*01, and most contained a 5-amino-acid CDRL3, a hallmark of V_H_1-2-encoded bnAbs to the CD4bs^8^ (Fig. 2a and Supplementary Table 3). However, due to the extensive level of hypermutation, the precise V_H_ allele assignment was ambiguous for some nAbs (for example, 04_A06 had 61.7%, 61.4% and 61.1% sequence identity to V_H_1-2*07, V_H_1-2*02 and V_H_1-2*04, respectively, as determined by the ImMunoGeneTics (IMGT) algorithm^18^; Fig. 2a and Supplementary Table 3). Isolated V_H_1-2-encoded antibodies were highly mutated, with 60–83% V_H_ gene germline nucleotide identities (Fig. 2a and Supplementary Table 3). Isolated heavy chain sequences of nAbs varied in length, position and/or sequence of insertions within FWRH1 or FWRH3, as well as in lengths and/or sequences of the CDRH3 (Fig. 2a and Supplementary Table 3). Whereas some nAbs lacked amino acid insertions, others displayed six- and four-amino-acid insertions in FWRH1 and FWRH3 or an ultralong insertion of 10 or 11 amino acids in FWRH1 (Fig. 2a and Supplementary Table 3), indicating parallel B cell evolution from distinct B cell progenitors. Clonal inference was challenging due to high mutation frequencies and the lack of widely accepted criteria for B cell clone differentiation. Therefore, we quantified sequence similarity as the fraction of shared V_H_ mutations and compared intradonor and interdonor pairs of nAbs encoded by the same V_H_ gene, using *IGHV1-2*-encoded bnAbs to the CD4bs from unrelated donors as a reference for unrelated sequences. nAb pairs within a donor that were as dissimilar as interdonor pairs were classified as different B cell clones from distinct progenitors. In donor EN02, intradonor similarities were bimodally distributed (Fig. 2b). A threshold defined between peaks separated clonally related from clonally unrelated sequences and revealed three clusters of distinct B cell clones with shared CD4bs specificity (Fig. 2b).

**Extended Data Fig. 4:**
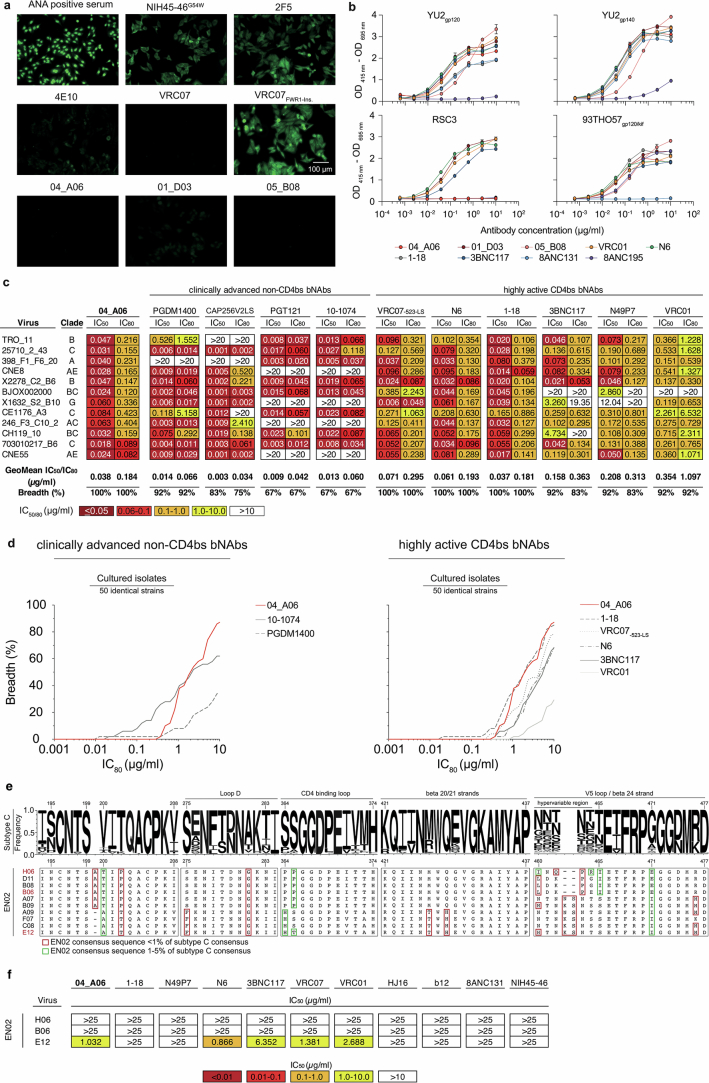
Autoreactivity and antiviral activity of isolated bNAbs from donor EN02. **a**, Reactivity of indicated antibodies against HEp-2 cells. Antibodies were tested at a concentration of 100 µg/ml in a single experiment. Data is presented from a single experiment. **b**, Binding profiles of isolated bNAbs from donor EN02 against indicated HIV-1 Env antigens. Samples were measured in technical duplicate within a single experiment, and data are presented as mean ± SD. **c**, Neutralization activity of bNAb 04_A06 and reference bNAbs against the HIV-1 global pseudovirus panel. Samples were tested in duplicates. Neutralization data of reference bNAbs were retrieved from CATNAP database^49^. **d**, Neutralization coverage (%) and potency (IC_80_) of 04_A06 against a panel of 50 donor-derived bulk outgrowth isolates. Neutralization data of reference bNAbs against replication competent viruses were retrieved from^10^. Samples were tested in duplicates. **e**, Amino acid frequency at selected sites across 744 clade C sequences from the Los Alamos National Laboratory (LANL) HIV database is shown (top; letter height reflects frequency). The lower panels depict corresponding positions in plasma-derived single genome sequencing (SGS) *env* sequences from donor EN02. Red and green boxes highlight amino acid residues present in <1% and 1–5%, respectively, of LANL clade C sequences. Numbering corresponds to HIV-1 HXB2 reference strain. **f**, Neutralization sensitivity of selected pseudoviruses carrying EN02 *env* sequences shown in **e**.

V_H_1-2-derived nAbs from expanded B cell clones were tested for BG505_SOSIP.664_ binding and neutralizing activity against the HIV-1 global pseudovirus panel^15^. Competed binding activity with other *IGHV1-2*-encoded bnAbs to the CD4bs suggested overlapping CD4bs specificity (Extended Data Fig. 3c). Except for one mAb, all evaluated antibodies bound soluble BG505_SOSIP.664_ and displayed neutralizing activity against the global pseudovirus panel (Fig. 2c). Potency and breadth were associated with mutation frequency and heavy chain amino acid insertions (Fig. 2c and Extended Data Fig. 3d). Members of clones 9 and 11 achieved up to 100% breadth, whereas clone 7 members reached only 50% (Fig. 2c and Supplementary Tables 3 and 4). These results were confirmed for representative members of each clone against an extended panel of ≥245 pseudovirus strains (Extended Data Fig. 3d). Only clone 9 nAbs comprising 10- or 11-amino-acid insertions in FWRH1 displayed high activity against all VRC01-class HIV-1_YU2_ escape variants, whereas members of remaining clones neutralized few or no variants (Fig. 2c). Transfer of the 11-amino-acid insertion from 04_A06 to members of other isolated clones and *IGHV1-2*-encoded reference bnAbs to the CD4bs, such as 05_B08 and VRC07 (referred to as VRC07_FWR-Ins_), maintained activity against the global HIV-1 and/or 119 multiclade panel and enabled these chimeric antibodies to overcome VRC01-class escape variants in vitro (Extended Data Fig. 3e,f). However, VRC07_FWR-Ins_ failed to overcome rebound viruses emerging during VRC07 monotherapy in HIV-1_YU2_-infected mice (Extended Data Fig. 3g). Additionally, unlike 04_A06, VRC07_FWR-Ins_ displayed high autoreactivity (Extended Data Fig. 4a). These findings suggested that although the identified B cell clones shared CD4bs specificities, they differed in unique genetic adaptations, such as ultralong FWRH1 amino acid insertions, which contributed to restriction of VRC01-class viral escape.

### 04_A06 has remarkable breadth and potency

Next, we determined the binding profiles of 04_A06, a mAb from clone 9, and VRC01-class bnAbs to the CD4bs against Env-derived proteins. Whereas both 04_A06 and VRC01-class antibodies were reactive to YU2_gp120_, 93THO57_gp120/kif_ and YU2_gp140_, 04_A06 did not bind to the V1–V3 loop-deficient resurfaced stabilized gp120 core 3 (RSC3) that was previously applied to selectively enrich VRC01-class antibodies^9,11^ (Extended Data Fig. 4b), implying a distinct mode of epitope recognition.

**Fig. 3:**
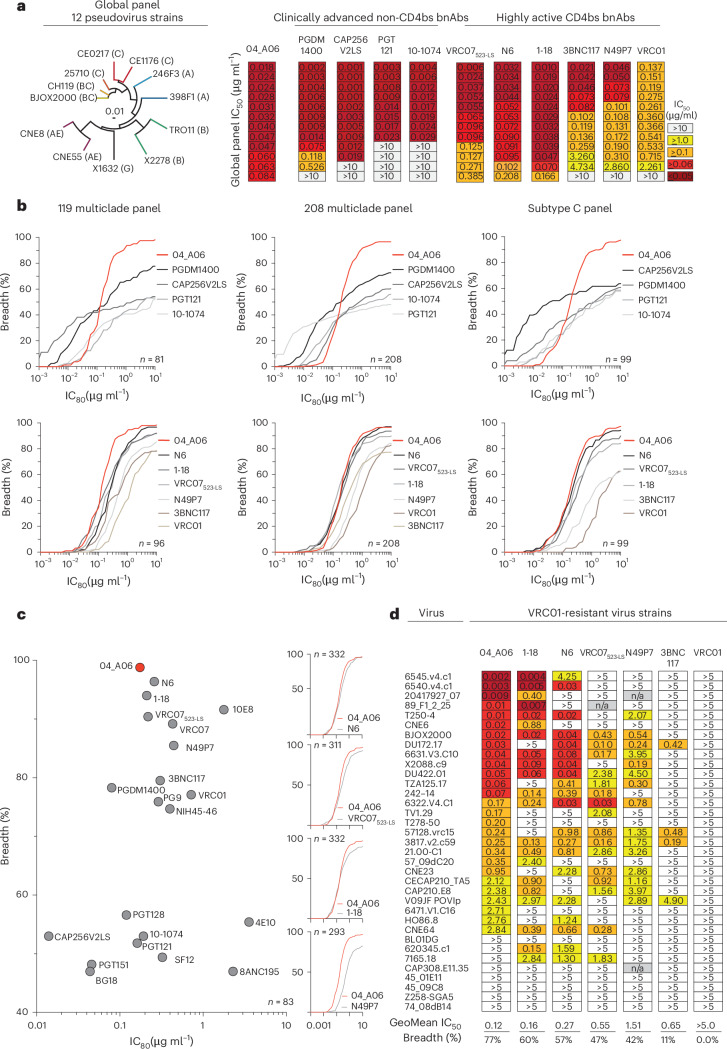
04_A06 demonstrates marked neutralizing potency and breadth. **a**, Phylogenetic tree depicting the distribution of Env-pseudotyped viruses of the 12-strain global panel^15^ (left) and heat maps illustrating neutralizing potency (IC_50_) of 04_A06 against the global panel compared to bnAbs not targeting the CD4bs currently investigated in advanced clinical trials (PGDM1400, CAP256V2LS, PGT121 and 10-1074) and to highly active bnAbs to the CD4bs (VRC07_523-LS_, N6, 1-18, 3BNC117, N49P7 and VRC01; right). Illustrated IC_50_ values for each bnAb are sorted by increasing value. Data for reference bnAbs were obtained from the CATNAP database^49^. 04_A06 samples were tested in duplicate in three independent experiments. GeoMean neutralization data are presented. **b**, Curve graphs illustrating neutralization coverage (%) and potency (IC_80_) of 04_A06 against multiclade panels of 119 (ref. ^19^) and 208 (ref. ^20^) pseudoviruses and a clade C pseudovirus panel compared to clinically advanced non-CD4bs (PGDM1400, CAP256V2LS, PGT121 and 10-1074; top) and highly active bnAbs to the CD4bs (1-18, N6, VRC07_523-LS_, N49P7, 3BNC117 and VRC01; bottom). Data are shown for identical virus strains from each panel for which reference neutralization data were available (*n*). Breadth (%) was defined applying a cutoff of ≤10 µg ml^−1^. **c**, Dot plot illustrating the neutralizing activity (IC_80_, breadth) of 04_A06 compared to HIV-1 bnAbs against 83 pseudoviruses and curve graphs comparing the neutralizing activity of 04_A06 to current highly active bnAbs to the CD4bs (N6, VRC07_523-LS_, 1-18 and N49P7) against panels of 332, 311, 332 or 293 pseudoviruses as in **b**. Neutralization data of bnAbs N6, 1-18 and 04_A06 were determined in the same laboratory. **d**, Neutralization profile (IC_50_, breadth) of 04_A06 compared to highly active bnAbs to the CD4bs (1-18, N6, VRC07_523-LS_, N49P7, 3BNC117 and VRC01) against a panel of 35 pseudoviruses resistant to VRC01. Data for reference bnAbs were obtained from the CATNAP database^49^, if available, otherwise bnAbs were tested in parallel to 04_A06. Reference antibodies were validated for functionality in neutralization assays against the global HIV-1 pseudovirus panel, and only those with IC_50_/IC_80_ values deviating less than threefold from CATNAP reference data^49^ were included. Breadth (%) was defined applying a cutoff of ≤5 µg ml^−1^. Samples were tested in duplicate in single experiments. GeoMean neutralization data are presented.

The antiviral activity of 04_A06 was then assessed against diverse reference HIV-1 pseudovirus panels comprising a total of 337 viral strains and against 50 outgrowth culture-derived primary HIV-1 isolates. 04_A06 neutralized 100% of the 12-strain HIV-1 global panel^15^ with higher potency (geometric mean (GeoMean) half-maximal inhibitory concentration/80% inhibitory concentration (IC_50_/IC_80_) = 0.038/0.184 µg ml^−1^) and/or breadth than clinically advanced bnAbs and previously identified bnAbs to the CD4bs (Fig. 3a, Extended Data Fig. 4c and Supplementary Table 4). Against well-established large multiclade pseudovirus panels, 04_A06 exhibited comparable neutralizing activity to previously described bnAbs to the CD4bs (Fig. 3b and Supplementary Table 5–7). 04_A06 displayed high potency (GeoMean IC_50_/IC_80_ = 0.037/0.135 µg ml^−1^) and breadth (98.3%/98.3%) against a 119-strain multiclade panel^19^ (Fig. 3b and Supplementary Table 5). In addition, 04_A06 demonstrated marked potency and neutralization breadth (GeoMean IC_50_/IC_80_ = 0.077/0.198 µg ml^−1^; breadth = 98.6%/96.6%) against a 208-strain multiclade panel^20^ (Fig. 3b and Supplementary Table 6) and high activity against a 100-strain clade C panel^21^ (GeoMean IC_50_/IC_80_ = 0.057/0.192 µg ml^−1^; breadth = 99%/98%; Fig. 3b and Supplementary Table 7). Only 5 or 9 strains across all 337 pseudovirus reference strains were resistant against 04_A06 at an IC_50_/IC_80_ ≥ 10 µg ml^−1^ (GeoMean IC_50_/IC_80_ = 0.059/0.176 µg ml^−1^; breadth = 98.5/97.3%; Fig. 3b,c and Supplementary Table 5–7). We also determined the activity of 04_A06 against replication-competent donor-derived outgrowth viruses, which are more challenging to neutralize than pseudoviruses^22^. 04_A06 levels of breadth and/or potency against viruses obtained from 50 PLWH were comparable to highly active bnAbs to the CD4bs (GeoMean IC_50_/IC_80_ = 0.45/1.736 µg ml^−1^; breadth = 94%/88%; Extended Data Fig. 4d and Supplementary Table 8). Pseudoviruses derived from plasma single-genome sequencing (SGS) *env* sequences of donor EN02 that comprised rare amino acid residues or insertions exhibited resistance to 04_A06 and other bnAbs to the CD4bs (Extended Data Fig. 4e,f). Finally, against a panel of 35 VRC01-resistant virus strains, near-pan-neutralizing bnAbs to the CD4bs 1-18 (ref. ^10^) and N6 (ref. ^7^) neutralized 57% and 60% of viruses, respectively, whereas 04_A06 achieved 77% breadth with higher potency (GeoMean IC_50_ = 0.12 µg ml^−1^; Fig. 3d). Thus, testing against large panels of pseudo- and replication-competent viruses revealed high neutralizing activity of 04_A06 including VRC01-resistant viral strains.

### Eleven-amino-acid insertion enables CD4bs and adjacent protomer binding

**Fig. 4:**
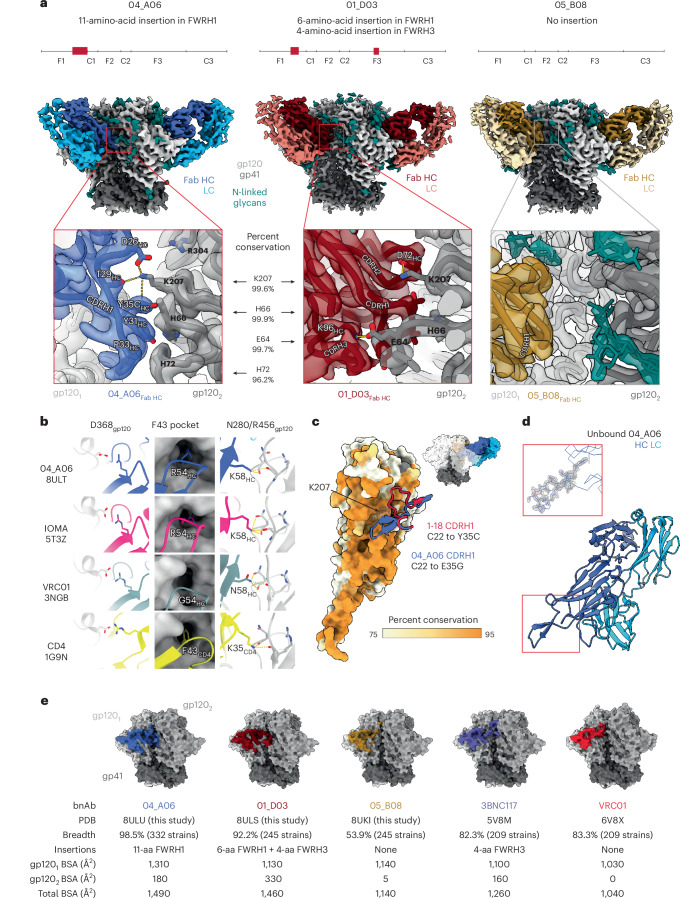
Ultralong 11-amino-acid insertion facilitates the quaternary binding mode of 04_A06. **a**, Schematics illustrating the position and length of FWRH1 and/or FWRH3 insertions of 04_A06 (left), 01_D03 (middle) and 05_B08 (right; top), EM maps showing side views of 04_A06, 01_D03 and 05_B08 bnAb Fabs in complex with BG505_SOSIP.664_ Env trimers (middle) and insets illustrating a close-up of bnAb interactions with the adjacent gp120 protomer (gp120_2_; bottom); HC, heavy chain; LC, light chain. **b**, Canonical interactions of CD4 and bnAbs to the CD4bs (04_A06, IOMA and VRC01) with Env gp120 D368, the F43 pocket and gp120 N280/R456. **c**, Interactions between the CDRH1s of 04_A06 (blue cartoon representation) and 1-18 (red cartoon representation) with the secondary (gp120_2_) Env protomer, shown as a surface colored by percent conservation. The inset (top right) shows the Env trimer–04_A06 complex as a surface representation for orientation. **d**, Crystal structure of unbound 04_A06 Fab (bottom) and inset highlighting the 04_A06 CDRH1, with electron density contoured at 1.5*σ* (top). **e**, Molecular surface representation showing the surface area buried by VRC01-class bnAbs (04_A06, 01_D03, 05_08, 3BNC117 and VRC01) on primary (gp120_1_) or secondary (gp120_2_) Env protomers (top). Listing of Protein Data Bank (PDB) accession codes, breadth (%), heavy chain insertion length and position, buried surface area (BSA) on gp120_1_ and gp120_2_ as well as total BSA for VRC01-class bnAbs (bottom).

**Extended Data Fig. 5:**
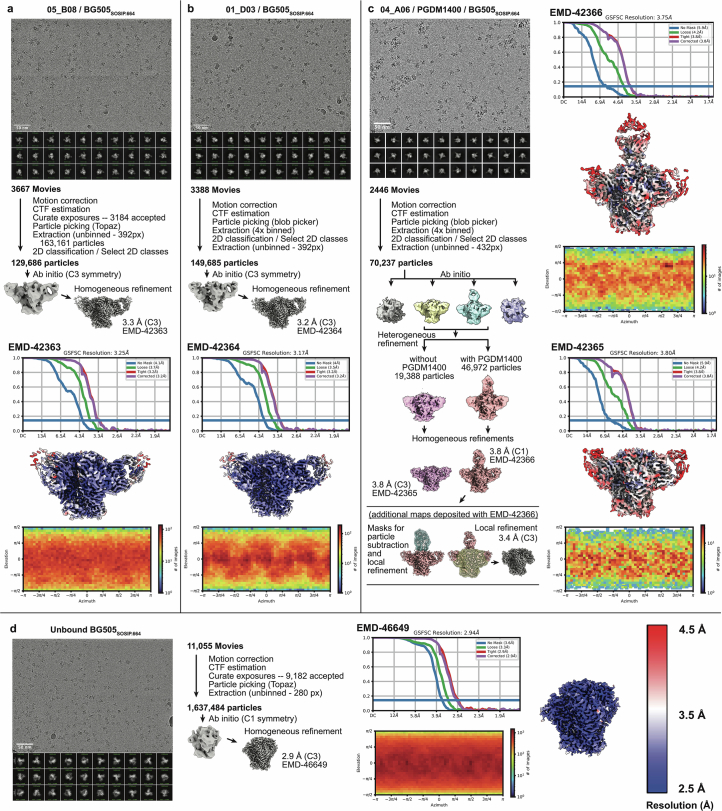
Cryo-EM data collection and processing. Representative micrographs, two-dimensional class averages, workflows, gold-standard Fourier shell correlation (GSFSC) curves, final density maps colored by local resolution, and particle orientation distributions for **a**, 05_B08 Fab / BG505_SOSIP.664_, **b**, 01_D03 Fab / BG505_SOSIP.664_, **c**, 04_A06 Fab / PGDM1400 Fab / BG505_SOSIP.664_, and **d**, unbound BG505_SOSIP.664_ datasets.

**Extended Data Fig. 6:**
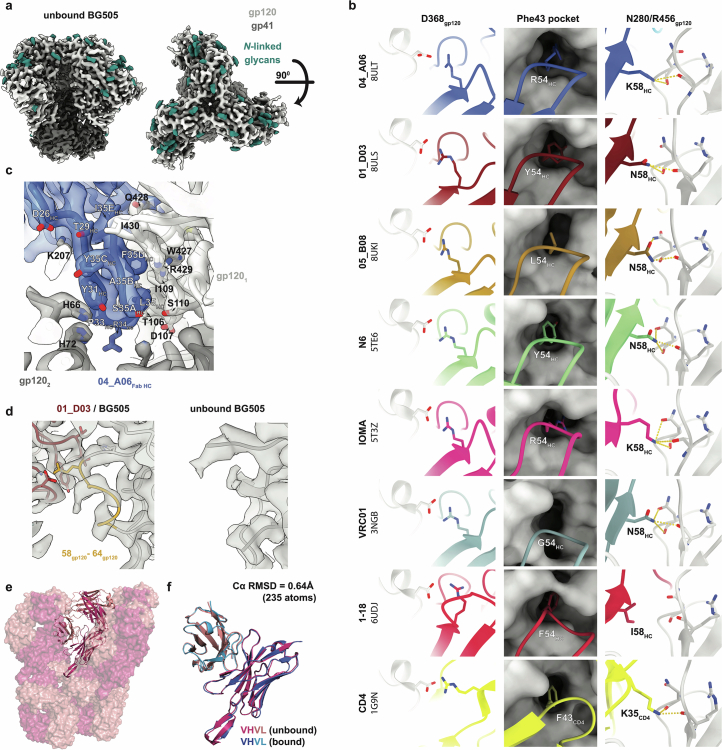
Structural analysis of CD4bs antibodies from EN02 with other CD4bs antibodies and unbound BG505. **a**, EM density of unbound BG505_SOSIP.664_ structure showing side and top views. **b**, Canonical interactions of CD4 and CD4bs bNAbs with Env: D368_gp120_, Phe43 pocket, and N280_gp120_ / R456_gp120_. **c**, Close up view of 04_A06’s CDRH1 interactions (blue) with the primary gp120 (gp120_1_). **d**, EM density for gp120 residues 58-64 on the adjacent protomer (gp120_2_) as observed in complex with 01_D03 Fab compared to unbound BG505_SOSIP.664_. **e**, Crystal packing environment of 04_A06 Fab. **f**, Overlay of V_H_-V_L_ domains in bound (from Fab-SOSIP cryo-EM structure) and unbound (from Fab crystal structure) structures.

To elucidate mechanisms of Env recognition, we determined single-particle cryo-electron microscopy (cryo-EM) structures of a BG505_SOSIP.664_ trimer^23^ unbound (2.9 Å) and in complex with representative Fabs from each expanded B cell clone: 04_A06 (3.8 Å), 01_D03 (3.2 Å) and 05_B08 (3.3 Å; Fig. 4a, Extended Data Figs. 5 and 6a and Supplementary Table 9). The 04_A06 complex also included a Fab from PGDM1400, a broader and more potent variant of the V_1_V_2_ bnAb PGT145 (ref. ^24^), and structures both with and without bound PGDM1400 were determined (Extended Data Fig. 5). All three bnAbs to the CD4bs exhibited canonical gp120 contacts made by CD4 and CD4-mimetic bnAbs^12,25,26^ (Fig. 4b and Extended Data Fig. 6b). In each structure, R71 of the heavy chain contacts D368 of gp120 (Fig. 4b and Extended Data Fig. 6b), a feature typical of VRC01-class antibodies and a hallmark of CD4-mimetic bnAbs^12^. Additionally, the V_H_1-2-encoded S54 of the heavy chain is mutated to R54 in 04_A06 and Y54 in 01_D03, which insert into a hydrophobic gp120 cavity analogous to CD4 residue F43 (ref. ^27^; Fig. 4b and Extended Data Fig. 6b). These interactions are similar to the interactions made by heavy chain R54 of the V_H_1-2-encoded, non-VRC01-class bnAb IOMA^26^ and heavy chain Y54 of the VRC01-class bnAb N6 (ref. ^7^; Fig. 4b and Extended Data Fig. 6b). Additionally, the V_H_1-2-encoded heavy chain N58 is mutated to K58 in 04_A06 and IOMA (Fig. 4b and Extended Data Fig. 6b), mimicking the interaction of CD4 K35 with Env residues N280 and R456 of gp120. Although 04_A06 features a five-amino-acid-long CDRL3 characteristic of VRC01-class bnAbs, it also shares key somatic hypermutations and structural determinants with IOMA shaping recognition of the CD4bs (Fig. 4b and Extended Data Fig. 6b). Thus, 04_A06 possesses features representative of both VRC01-class and IOMA-like bnAbs to the CD4bs.

The presence and length of insertions in the three representative bnAbs correlated with the formation of interprotomer contacts (Fig. 4a). 04_A06 and 01_D03, but not 05_B08, contacted the adjacent gp120 protomer (Fig. 4a), a feature seen in other potent bnAbs such as 1-18 (ref. ^10^) and 3BNC117 (ref. ^28^). Contacts between 04_A06 and the adjacent protomer were mediated exclusively via a protruding CDRH1 in 04_A06, a consequence of the 11-residue FWRH1 insertion (Fig. 4a), which also contacted the primary protomer (Extended Data Fig. 6c). On the adjacent protomer, 04_A06 interacted with gp120 K207 (99.6% conserved; www.hiv.lanl.gov and West et al.^29^) and formed a potential electrostatic interaction with heavy chain D26 (Fig. 4a). D26 is also positioned adjacent to gp120 R304 (93.5% conserved), whereas residues within the tip of the 04_A06 CDRH1 (heavy chain Y31–Y35C) are in close proximity to residues H66 and H72 on gp120 (99.9% and 96.2% conserved, respectively; Fig. 4a). Together, these residues could form interactions that permit tolerance to structural variability within the CD4bs on the primary Env protomer (for example, the addition of a potential N-linked glycosylation site at gp120 N279). The extended CDRH1 of the CD4bs bnAb 1-18 also contacts gp120 K207 on the adjacent protomer but mainly interacts with less conserved V3 residues^10^, whereas the CDRH1 of 04_A06 extends toward a more conserved gp120 region (Fig. 4c), likely contributing further to 04_A06’s enhanced neutralization profile. In contrast to the CDRH1-mediated interprotomer contacts of 04_A06, mAb 01_D03, with its 6-residue FWRH1 insertion, 4-residue FWRH3 insertion and 20-residue CDRH3, contacts the adjacent protomer with each of its heavy chain CDRs and makes potential electrostatic interactions with gp120 K207 and E64 (99.6% and 99.7% conserved; Fig. 4a). In the 01_D03-complexed Env structure, but not in the unbound BG505 SOSIP structure, EM density was observed for residues 57–65 of gp120 on the adjacent protomer (Extended Data Fig. 6d), suggesting that this normally disordered loop becomes stabilized after antibody binding.

To determine whether the conformation of the extended CDRH1 of 04_A06 is preorganized for binding, we solved a 1.75-Å crystal structure of unbound 04_A06 Fab (Fig. 4d and Supplementary Table 9). The structure, which does not appear to be influenced by crystal contacts (Extended Data Fig. 6e), revealed a well-ordered antibody combining site and CDRH1 that resembled the conformation of the combining site in the Env-bound Fab (root mean square deviation (r.m.s.d.) = 0.64 Å; 235 Cα atoms), consistent with a lock-and-key mechanism of binding^30^ (Fig. 4d and Extended Data Fig. 6f). This is in contrast to the induced fit mechanism of binding by the extended CDRH1 of VRC–CH31, which is unresolved in crystal structures in complex with gp120 (ref. ^31^) but becomes partially ordered in a VRC–CH31–SOSIP Env structure, where it is stabilized by interactions with the adjacent protomer^32^. The preorganized antibody combining site of 04_A06 likely leads to a more favorable interaction with Env due to a lower entropic cost of binding.

In addition to contacting highly conserved residues, the insertions and interprotomer contacts of 04_A06 and 01_D03 contribute to an increased amount of surface area buried on Env by antibody binding. These insertions enable 04_A06 and 01_D03 to bury more surface area on Env than 05_B08 or other VRC01-class bnAbs (Fig. 4e), presenting another possible mechanism contributing to breadth, potency and resistance to escape^10^. Consistent with this and in common with 04_A06, 01_D03 exhibited greater potency and breadth than 3BNC117 and VRC01 (GeoMean IC_50_/IC_80_ = 0.052/0.187 µg ml^−1^, breadth = 92%/88%; 245 strains; Fig. 4e).

**Extended Data Fig. 7:**
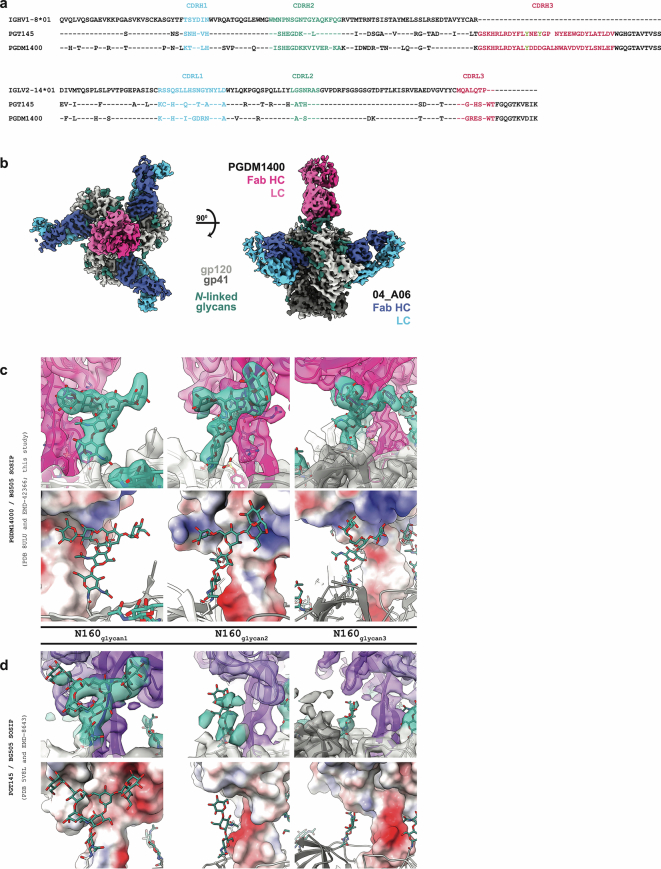
Structural analyses of PGDM1400. **a**, Amino acid sequence alignment of heavy and light chain V gene-encoded regions from bNAbs PGT145 and PGDM1400. Sulfated tyrosine residues located in the CDRH3 are indicated by a green “Y”. **b**, Structure overview showing EM density of BG505_SOSIP.664_ in complex with 04_A06 and PGDM1400 Fabs. EM density for N160 glycans (top) and a Fab electrostatic surface calculation (bottom) for PGDM1400 (**c**) and PGT145 (**d**). Glycans labeled as glycan 1,2, and 3 correspond to the N160_gp120_ glycans from each of the three Env protomers^28^.

Our 04_A06 complex structure also included PGDM1400 (Extended Data Fig. 7a). The PGDM1400 Fab binds asymmetrically to the trimer apex with a stoichiometry of one Fab per trimer (Extended Data Fig. 7b). Its 34-residue CDRH3 is inserted down the trimer symmetry axis and contacts protein residues and the gp120 N160 N-glycan of all three protomers, resulting in a well-resolved glycan density for the core pentasaccharide of each N160 glycan and additional glycans in some cases (Extended Data Fig. 7c). Mass spectrometry analysis reported that the gp120 N160 glycan is underprocessed, containing a mixture of high-mannose, hybrid and complex-type N-glycans^33^. In our structure, density was not observed for a core fucose, a component of complex-type N-glycans^26^, nor could clear density be discerned much beyond the core pentasaccharide (Extended Data Fig. 7c), the latter a likely consequence of limited resolution, glycan compositional heterogeneity and/or glycan flexibility. However, a surface electrostatic calculation revealed electropositive patches that could accommodate, or interact with, negatively charged sialic acid residues on complex-type glycans^34^ (Extended Data Fig. 7c), particularly for surfaces on PGDM1400 that interact with the N160 glycan 2 and N160 glycan 3 relative to analogous interactions with PGT145 (ref. ^28^; Extended Data Fig. 7c,d). Additionally, PGDM1400 appears to stabilize the core pentasaccharide of the N160 glycan 3 (Extended Data Fig. 7c,d), whereas this glycan was proposed to be inhibitory to the binding of PGT145 (ref. ^28^). The ability to accommodate and/or interact with the three gp120 N160 glycans on an Env trimer, whether or not processed beyond high-mannose carbohydrates, likely contributed to the enhanced breadth and potency of PGDM1400. Our structure recapitulates key molecular interactions between PGDM1400 and Env recently reported^35^, such as the interaction between gp120 K169 and heavy chain Tys100F, electrostatic interactions between an Asp-Asp-Asp motif at the tip of the PGDM1400 CDRH3 with gp120 R166 of all three protomers and extensive glycan density at gp120 N160 on all protomers.

### 04_A06 restricts escape and fully suppresses viremia in vivo

**Extended Data Fig. 8:**
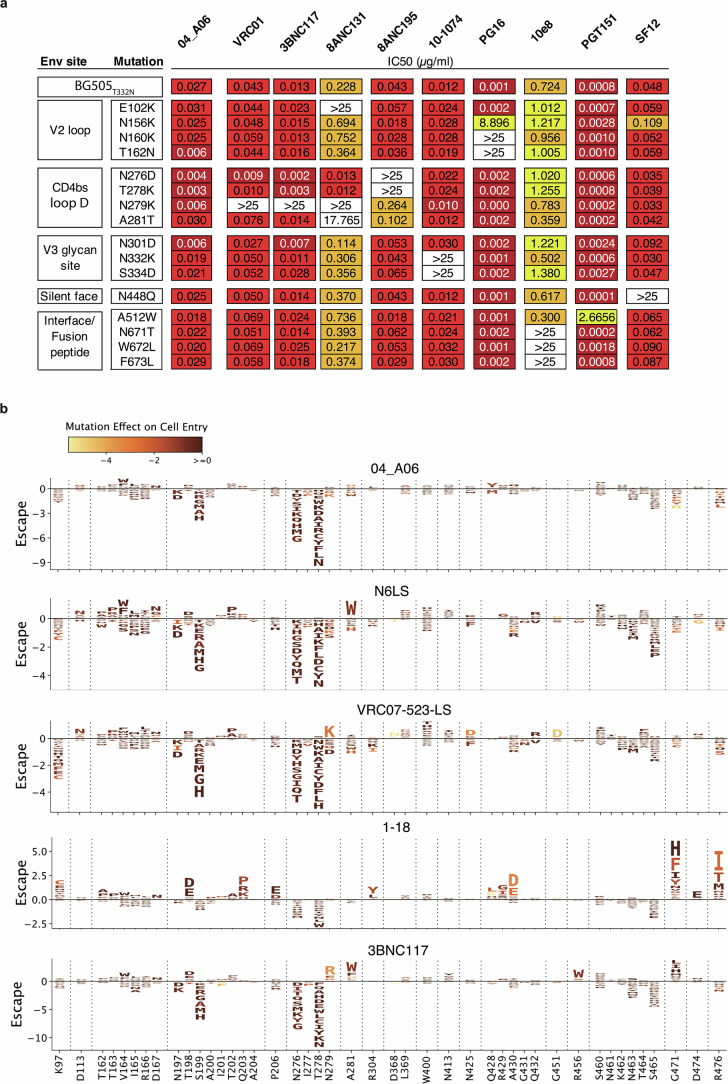
Restriction of viral escape *in vitro*. **a**, Neutralizing activity of 04_A06 against BG505_T332N_ and common escape pseudovirus mutants. The panels indicate the IC_50_ of 04_A06 against the respective pseudovirus variant. **b**, Logo plots illustrating the extent of neutralization escape caused by mutations in the HIV-1 Env strain BF520 for antibodies 04_A06, N6-LS, VRC07_-523-LS_, 1-18 and 3BNC117. The heights of individual letters represent the effect of that amino-acid mutation on antibody neutralization, with positive heights (letters above the zero line) indicating mutations that cause escape, and negative heights (letters below the zero line) indicating mutations that increase neutralization. Letters are colored by the effect of that mutation on pseudovirus cell entry, with yellow corresponding to reduced cell entry and brown corresponding to neutral effects on cell entry. The y-axis scales for each antibody are independent. Only key sites are illustrated. See https://dms-vep.org/HIV_Envelope_BF520_DMS_04-A06/htmls/04-A06_mut_effect.html, https://dms-vep.org/HIV_Envelope_BF520_DMS_04-A06/htmls/N6_LS_mut_effect.html, https://dms-vep.org/HIV_Envelope_BF520_DMS_04-A06/htmls/VRC07_523_LS_mut_effect.html, https://dms-vep.org/HIV_Envelope_BF520_DMS_04-A06/htmls/1-18_mut_effect.html, and https://dms-vep.org/HIV_Envelope_BF520_DMS_04-A06/htmls/3BNC117_mut_effect.html for interactive versions of the escape maps.

To determine viral escape pathways, we first assessed the antiviral activity of 04_A06 against a library of known HIV-1_BG505_T332N_ Env escape mutations^10^. None of the evaluated mutations, including loop D substitutions gp120 N279K and gp120 A281T that typically interfere with bnAbs to the CD4bs^6,10^, conferred resistance against 04_A06 (Extended Data Fig. 8a). Next, we applied lentivirus deep mutational scanning (DMS)^36,37^ to comprehensively measure how all functionally tolerated Env_BF520_ mutations affected neutralization by 04_A06 and CD4bs reference bnAbs (Extended Data Fig. 8b). Env mutations in loop D caused escape from bnAbs N6LS (A281W and A281T), VRC07_523-LS_ (N279K) and 3BNC117 (N279R/N279E/N279K and A281W/A281F/A281D/A281I/A281V; Extended Data Fig. 8b). Mutations in the β23/V5 loop caused escape from N6LS (G451D) and 3BNC117 (R456W/R456S and G471; Extended Data Fig. 8b). Substitutions potentially reducing neutralization sensitivity of 1-18 were identified in gp120 at sites 198, 203 and 206 near the N197 glycosylation motif and V2 loop, 428–430 in the β20/β21 regions and 471, 474 and 476 in the β23/V5 loop regions (Extended Data Fig. 8b). DMS did not identify any single mutation to Env_BF520_ that strongly escaped 04_A06, whereas few mutations (for example, V164W and Q428Y) only slightly reduced neutralization sensitivity (Extended Data Fig. 8b).

**Fig. 5:**
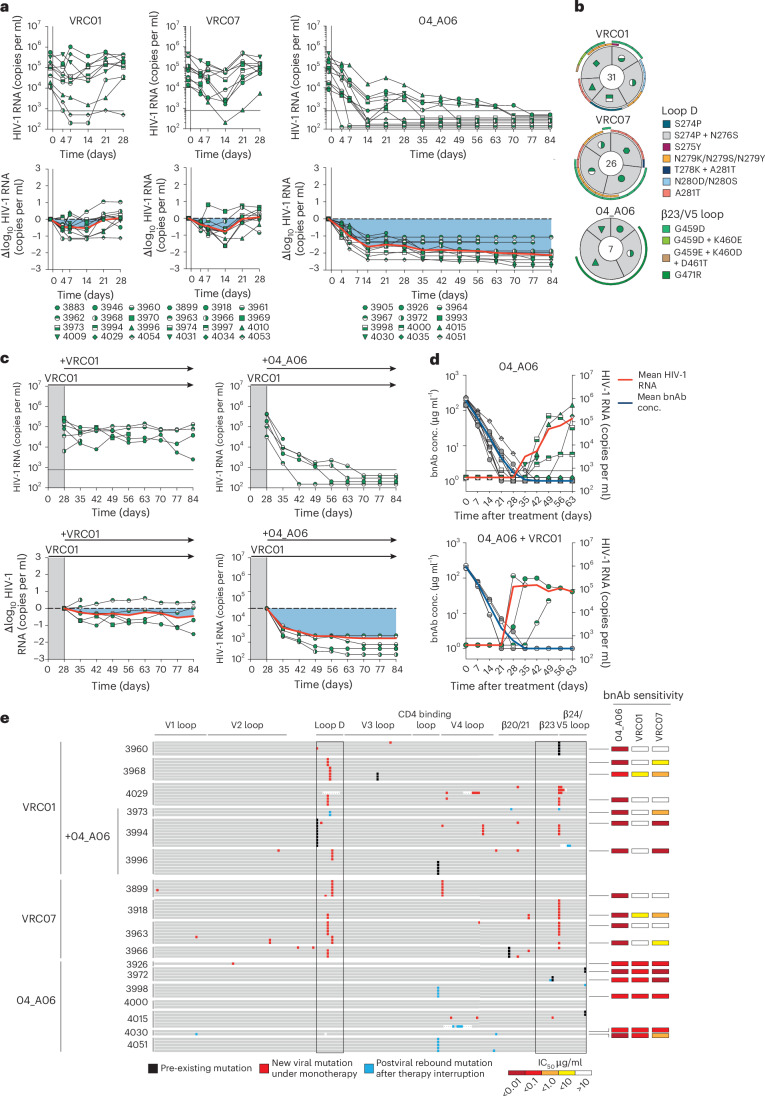
04_A06 suppresses viremia and restricts VRC01-class viral escape in vivo. **a**, Curve graphs showing the absolute HIV-1 RNA plasma copies per ml (top) and relative log_10_ changes from baseline viral loads (bottom) after initiation of bnAb therapy with VRC01, VRC07 and 04_A06 in HIV-1_YU2_-infected humanized NOD.Cg-*Rag*^1tm1mom^*Il2rg*^tm1Wjl^/SzJ (NRG) mice (*n* = 36). Gray lines (top graphs) indicate the LLQ of the qPCR assay (784 copies per ml), and red lines indicate the average log_10_ changes compared to baseline viral loads (day –1). **b**, Pie charts showing the proportion of identified amino acid substitutions among all single-genome plasma HIV-1 *env* sequences generated from HIV-1_YU2_-infected humanized mice at day 28 after initiation of treatment with VRC01, VRC07 and 04_A06 as in **a**. The total number of analyzed sequences is indicated in the center of each pie chart. Mice are labeled according to icon legends as in **a**. Colored bars on the outside of the pie charts indicate mutations in loop D and β23/V5 loop. **c**, Curve graphs illustrating plasma viral load dynamics in HIV-1_YU2_-infected humanized NRG mice treated with VRC01 (0.5 mg) in combination with VRC01 (0.5 mg, *n* = 6, left) or 04_A06 (0.5 mg; right; *n* = 5) initiated at day 28 following viral rebound and continued until day 84 as in **a**. The red line indicates the average log_10_ change compared to baseline viral loads (day 28). Mice are labeled according to icon legends as in **a. d**, HIV-1 viral loads (right *y* axis) and plasma bnAb concentrations (left *y* axis) in HIV-1_YU2_-infected humanized NRG mice (*n* = 16) after treatment interruption on day 84 showing mean plasma bnAb concentration (gray icons) and GeoMean HIV-1 viral loads (green icons). The gray line indicates the LLQ of the qPCR assay (784 copies per ml). Mice are labeled according to icon legends as in **a**. **e**, Illustration of pre-existing (day –1), new viral mutations under bnAb monotherapy (day 28) and postviral rebound mutations after therapy interruption in the V1, V2 loop, loop D, V3 loop, CD4 binding loop, V4 loop, β20/21 and/or β24/V5 loop of SGS-derived *env* sequences (left) derived from the plasma of NRG mice immunized with VRC01 (alone or in combination with 04_A06 at day 28, as in **c**), VRC07 and 04_A06 and sensitivity of the respective pseudovirus (right). Samples were tested in duplicate in single experiments. GeoMean neutralization data are presented; conc., concentration.

**Extended Data Fig. 9:**
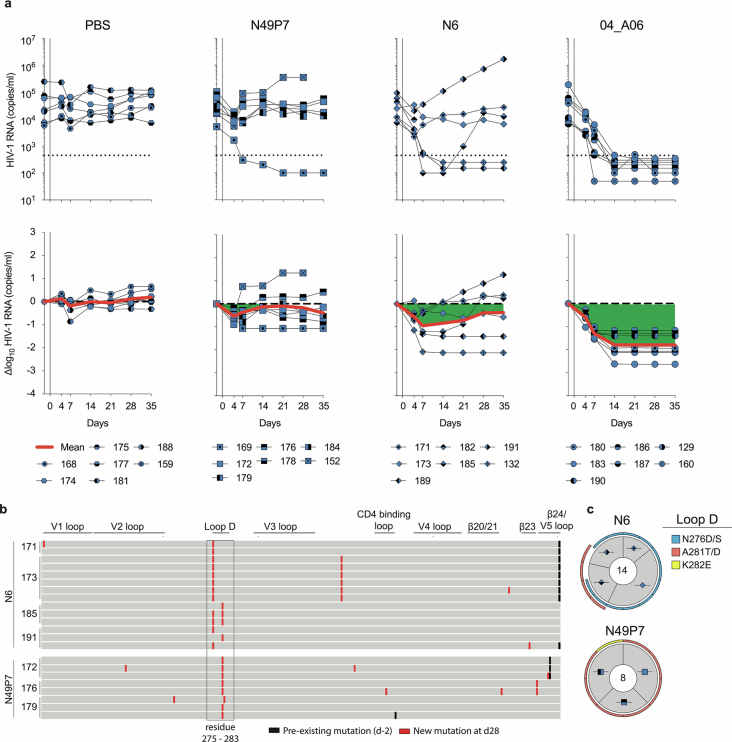
*In vivo* antiviral activity of 04_A06 in comparison to highly-active CD4bs bNAbs. **a**, Investigation of the antiviral activity of N49P7, N6, and 04_A06 monotherapy in HIV-1_YU2_-infected humanized mice (NXG-HIS) (n = 28). Graphs illustrate the absolute HIV-1 RNA plasma copies/ml (top) and relative log_10_ changes from baseline viral loads (bottom) after initiation of bNAb therapy. Dashed lines (top graphs) indicate the lower limit of quantitation of the qPCR assay (LLQ) (451 copies/ml). Red lines display the average log_10_ changes compared to baseline viral loads (day -2). **b**, Alignment of plasma SGS-derived *env* sequences identified from day -1 (black bars) and after viral rebound from day 28 (red bars). **c**, Analyses of single HIV-1 plasma *env* sequences from HIV-1_YU2_-infected humanized mice obtained on day 28 after bNAb treatment initiation. Total number of analyzed sequences is indicated in the center of each pie chart. Mice are labeled according to icon legends in a. Colored bars on the outside of the pie charts indicate mutations in Loop D.

To investigate in vivo antiviral activity, 04_A06 was administered subcutaneously (1 mg loading dose, 0.5 mg twice weekly) to HIV-1_YU2_-infected humanized mice for up to 12 weeks. PBS-treated mice used as untreated controls showed maintained viremia for ≥12 weeks (Extended Data Fig. 3g). Administration of bnAbs to the CD4bs (VRC01 and VRC07) induced transient viral load reductions (up to 0.54/0.8 log_10_ copies per ml at week 2 after treatment initiation), followed by viral rebound within 3 weeks of treatment initiation (Fig. 5a). N49P7 and N6 monotherapy caused temporary viral load suppression in 50–86% of mice (six of seven for N49P7; three of six for N6), with rebound occurring between 14 and 28 days after treatment initiation (Extended Data Fig. 9a). 04_A06 therapy decreased the mean HIV-1 viral load by up to 2.12 log_10_ copies per ml at week 12 after treatment initiation and maintained full suppression (lower limit of quantitation (LLQ) < 784 or 451 copies per ml) in 19 of 19 mice without viral rebound until week 12 (Fig. 5a, Extended Data Fig. 9a and Supplementary Table 10). SGS *env* sequencing of plasma virus from week 4 after treatment initiation revealed accumulation of mutations at Env gp120 residues N279, N280, A281 and G459 in the Env loop D and/or β23/V5 loop mediating resistance to VRC01 and VRC07, but not to 04_A06 (Fig. 5b and Supplementary Table 10). N49P7 and N6 monotherapy selected for mutations at gp120 residues N276, A281 and K282 (Extended Data Fig. 9b and Supplementary Table 10). gp120 A281 represents a key contact residue for N49P7 (ref. ^6^), and gp120 A281T was reported to mediate resistance against N6 (ref. ^10^). In contrast, we found no selection of mutations affecting 04_A06 sensitivity in the 04_A06-treated mice, before they achieved full suppression (Fig. 5b and Supplementary Table 10). To determine 04_A06 activity against VRC01-resistant viral variants in vivo, we administered 04_A06 to VRC01-pretreated mice following viral rebound (week 4 after treatment initiation) and continued VRC01 therapy in parallel with 04_A06 therapy (1 mg loading dose followed by 0.5 mg twice weekly for each antibody) to maintain VRC01 selection pressure. A double dose of VRC01 served as control. Although the double dose of VRC01 had no effect, the addition of 04_A06 led to complete suppression of viremia in all VRC01-pretreated animals for up to 8 weeks (Fig. 5c).

To determine whether waning antibody levels after treatment interruption promoted emergence of 04_A06 escape, we discontinued therapy and monitored mice for 9 weeks after discontinuation. The median duration to viral rebound was 38.5 days occurring after plasma antibody levels decreased below 3 µg ml^−1^ (04_A06 + VRC01) or were undetectable (04_A06; LLQ < 1 µg ml^−1^; Fig. 5d). Three of 13 mice showed no rebound of viremia at week 9 or at the time of death (Fig. 5d), potentially due to graft failure. SGS of plasma rebound virus *env* genes, together with limited functional characterization of derived pseudoviruses, revealed no consistent amino acid substitutions conferring resistance to 04_A06 across all 13 mice (Fig. 5e and Supplementary Table 10 and 11). We conclude that 04_A06 monotherapy effectively mediates long-term control of viremia in HIV-1_YU2_-infected humanized mice and could restrict and overcome VRC01-class viral escape in vitro and in vivo.

### In silico analyses predict potential of 04_A06 for prevention

**Fig. 6:**
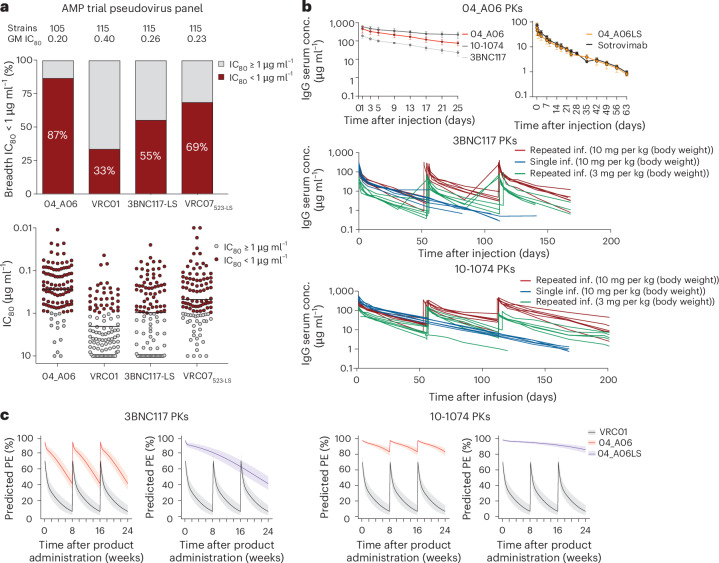
04_A06 exhibits enhanced antiviral activity against AMP trial viruses and high HIV-1 PE. **a**, Bar graphs showing the breadth (%) of 04_A06, VRC01, 3BNC117-LS and VRC07_523-LS_ (top) and dot plots indicating the IC_80_ values of bnAbs (bottom) against representative 105 (04_A06) or 115 (VRC01, 3BNC117-LS and VRC07_523-LS_) pseudoviruses generated from the placebo arms of the HVTN703/HPTN 081 trial in predominantly clade B regions and the HVTN704/HPTN 085 trial in predominantly clade C regions, respectively^13,38^, neutralized (IC_80_ < 1 μg ml^−1^) or not (IC_80_ ≥ 1 μg ml^−1^) at the established threshold of protection. Data for reference bnAbs were retrieved from the CATNAP database^49^. Antibodies were tested in duplicate in single experiments. The black lines indicate GeoMean. **b**, Curve graphs illustrating serum concentrations (µg ml^−1^) of 04_A06, 3BNC117 and 10-1074 at days 0–25 (top left) or 04_A06LS and sotrovimab at days 0–63 (top right) in hFcRn transgenic mice injected intravenously with a single dose of 0.5 mg of 04_A06, 3BNC117 or 10-1074 (*n* = 4 per group) or 5 mg per kg (body weight) of 04_A06LS or sotrovimab (*n* = 5 per group; top). Serum concentrations of 3BNC117 (middle) or 10-1074 (bottom) at days 0–200 in individuals who received a single infusion (10 mg per kg (body weight)) or three repeated infusions of 3 mg per kg (body weight) or 10 mg per kg (body weight; every 8 weeks) as part of a human clinical trial^40^. Each dot represents the mean ± s.d. serum concentration for all mice in the group at a given time point, with individual serum samples measured in technical duplicate by enzyme-linked immunosorbent assay (ELISA); inf., infusion. **c**, Curve graphs showing the predicted HIV-1 PE of 04_A06, 04_A06LS or VRC01 at weeks 0–24 after three infusions of 04_A06 every 8 weeks or a single infusion of 04_A06LS (all 30 mg per kg (body weight)) assuming 3BNC117 PKs^40^ (left) or 10-1074 PKs (right). Predictions for 04_A06-LS assumed that 04_A06-LS has a 2.5-fold longer half-life than 04_A06. Solid lines display the median and shaded areas display the 95% prediction interval.

**Extended Data Fig. 10:**
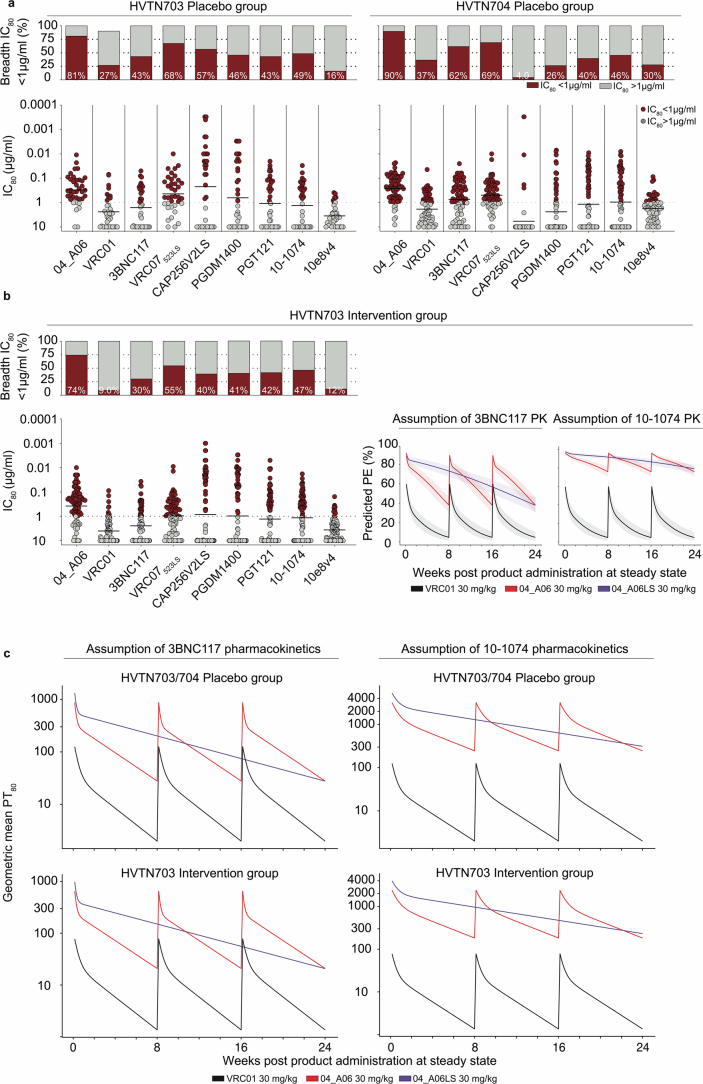
Neutralization profile and predicted PE against AMP trial pseudoviruses. Antiviral activity of 04_A06 and reference bNAbs against pseudoviruses generated from **a**, the HVTN703 and HVTN704 placebo group and **b**, HVTN703 intervention group, respectively. Bar graphs display the breadth (%) at the established threshold of protection (IC_80_ ≤ 1 µg/ml). Dot plots illustrate the potency of tested bNAbs (IC_80_) against each pseudovirus strain. Antibodies were tested in duplicate within single experiments. Neutralizing activity of mAbs was measured against 37 HVTN703 and 68 HVTN704 placebo group viruses, and 86 HVTN703 intervention group viruses. Black lines in the dot plots indicate the geometric mean IC_80_ values. Predicted HIV-1 PE of 04_A06 over time against HVTN703 intervention group pseudoviruses in comparison to VRC01. Left graph illustrates the PE of 04_A06 (red) and 04_A06LS (purple) under the assumption of 3BNC117 pharmacokinetics. The right graph displays the PE of 04_A06 and 04_A06LS under the assumption of 10-1074 pharmacokinetics. Black curves indicate the VRC01 PE in the AMP trials. Solid lines display the median and shaded areas the 95% prediction interval. **c**, Illustration of the geometric mean PT_80_ against placebo and intervention group viruses. The geometric mean PT_80_ for each time point was determined by dividing the geometric mean of the predicted serum concentration of bNAbs in recipients at each time point during steady state (as simulated via PK modeling for each bNAb, as outlined in the methods section) by the geometric mean IC_80_ of the bNAb against viruses present in the specified AMP trial. Calculations were performed over time after three 8-weekly infusions of 04_A06 at 30 mg/kg or a single infusion of 04_A06LS at 30 mg/kg. In the scenario involving the LS-modified version, predictions were made under the premise that 04_A06LS has a 2.5-fold higher half-life than 04_A06. Black lines indicate the PT_80_ for VRC01. Data for reference bNAbs were retrieved from CATNAP database.

To assess the potential of 04_A06 for prevention, we determined its activity against AMP trial-derived HIV-1 pseudoviruses representing contemporaneous circulating HIV-1 strains. These pseudoviruses were generated based on *env* sequences observed after HIV-1 breakthrough infections from placebo arms (HVTN703/HPTN 081 and HVTN704/HPTN 085) or under VRC01 selection pressure (HVTN703/HPTN 081 trial)^13^. Most pseudoviruses belonged to clade C (HVTN703/HPTN 081) or clade B (HVTN704/HPTN 085)^13^. 04_A06 displayed high levels of potency and breadth against both placebo (GeoMean IC_50_/IC_80_ = 0.07/0.26 µg ml^−1^; breadth = 100%/98%, at ≤10 µg ml^−1^) and HVTN703 treatment group viruses (GeoMean IC_50_/IC_80_ = 0.10/0.31 µg ml^−1^; breadth = 97%/94%, at ≤10 µg ml^−1^; Fig. 6a and Extended Data Fig. 10a,b). Applying the AMP trial protection threshold of IC_80_ < 1 µg ml^−1^ (refs. ^13,38^), 04_A06 exhibited a breadth of 87% (GeoMean IC_80_ = 0.20 µg ml^−1^, at <1 µg ml^−1^) against placebo and 74% (GeoMean IC_80_ = 0.18 µg ml^−1^ at <1 µg ml^−1^) against the HVTN703 treatment group viruses (Fig. 6a and Extended Data Fig. 10a,b), whereas VRC01 neutralized only 33% and 9.3%, respectively. (Fig. 6a). Notably, 04_A06 also remained active against HVTN703 treatment group viruses that exhibited high resistance to VRC01 (Extended Data Fig. 10b and Supplementary Table 12).

Next, we modeled the HIV-1 prevention efficacy (PE) of 04_A06 applying the predicted serum neutralization 80% inhibitory dilution titer (PT_80_; Extended Data Fig. 10c), established as a correlate of protection^38^ integrating the in vitro neutralization potency (IC_80_) and pharmacokinetic (PK) profile of a bnAb^38^. The PKs of 04_A06 were assessed in human FcRn transgenic mice, which mirror antibody PKs in humans^39^. Following a single intravenous injection, 04_A06 displayed a PK profile between 3BNC117 and 10-1074 (ref. ^40^; Fig. 6b). The LS-engineered (M428L, N434S) variant (04_A06_LS) closely resembled the PKs of sotrovimab^41^, a mAb directed against severe acute respiratory syndrome coronavirus 2 (SARS-CoV-2) with enhanced hFcRn binding and extended half-life. We hypothesized that the PK profile of 04_A06 parallels 3BNC117 or 10-1074 in humans and modeled the PT_80_ and PE of 30 mg per kg (body weight) 04_A06 under the assumption of either 3BNC117-like or 10-1074-like PKs^40^ (Fig. 6b,c). Simulations based on PK modeling for 04_A06 administered at 8-week intervals for 24 weeks (refs. ^38,42^) yielded a time-averaged mean predicted PE (mPE) of 66.5% or 90.9% assuming 3BNC117 or 10-1074 PKs, which exceeded the 23.3% mPE of VRC01 (Fig. 6c). PK predictions for a single administration of 04_A06_LS (Fig. 6c), considering a conservative 2.5-fold increase in the elimination half-life^43^, revealed 79.1% or 93.1% mPE for a period of 6 months (Fig. 6c). In conclusion, 04_A06 demonstrates remarkable antiviral efficacy against pseudoviruses derived from the AMP trials, suggesting it may be a promising antibody candidate for future prevention strategies.

## Discussion

In this study, we identified bnAb 04_A06 through detailed analysis of 32 HIV-1 elite neutralizers. 04_A06 showed high activity against AMP trial and traditional pseudovirus panels. Furthermore, in vitro neutralization assays against resistant isolates, DMS and in vivo experiments indicated that 04_A06 effectively limited HIV-1 escape.

DMS of VRC01-class antibodies has revealed escape involving canonical sites in loop D (gp120 N279 and A281), glycan-associated mutations near gp120 N197 and distal sites like gp120 I326 (ref. ^44^). Our results align with these findings, as we observed escape from VRC01-class antibodies N6LS, VRC07_523-LS_ and 3BNC117 via mutations at gp120 N279 and/or A281, whereas 1-18 was affected by mutations near the N197 glycosylation motif. 04_A06 differed markedly in that no single mutation conferred strong escape in DMS. However, our DMS analyses were conducted using a single pseudovirus strain (HIV-1_BF520_) and may not reflect diversity or evolutionary dynamics of replication-competent viral swarms. Nonetheless, in HIV-1_YU2_-infected humanized mice, 04_A06 achieved complete and sustained viral suppression extending up to 28 days beyond treatment interruption. 04_A06 overcame VRC01-class-resistant viruses in pretreated mice and fully suppressed viremia in all animals. Compared to bnAb 1-18, which also demonstrated prolonged suppression of viremia in vivo, 04_A06 displayed superior activity against strains resistant to bnAbs to the CD4bs and a unique escape profile identified through DMS^10^, which suggested that 04_A06 imposed strong selection pressure and fitness costs on HIV-1. Our in vivo studies were conducted using a single virus strain (HIV-1_YU2_) and are limited to fully recapitulate viral diversity in natural infection.

Structural analysis of 04_A06 revealed mechanisms that contributed to the breadth and potency of the antibody. Most notably, 04_A06 uses an ultralong 11-amino-acid insertion in FWRH1 that contacts gp120 K207, H66 and H72, highly conserved residues on the adjacent gp120. Pseudoviruses containing substitutions at these residues exhibit decreased or completely abrogated infectivity^32^, suggesting that these residues are functionally important and that escape mutations at these sites are likely associated with fitness costs. Engrafting the FWRH1 insertion from 04_A06 to VRC07 restored neutralizing activity against VRC01-class-resistant viruses in vitro, supporting the functional relevance of these contacts for restriction of viral escape. To our knowledge, among the antibodies identified to date, only 04_A06 contacts this conserved surface on HIV-1 Env, a region that is sterically difficult for antibody access. In addition, an unliganded 04_A06 Fab structure showed that its FWRH1 insertion is preorganized for binding, despite potential flexibility arising from it extending away from the antibody combining site, suggesting no entropic penalties for reorganizing the FWRH1 insertion after Env binding. Beyond these heavy chain features, 04_A06 diverges from canonical V_H_1-2-encoded bnAbs to the CD4bs in light chain architecture. Unlike VRC01 or VRC07, which exhibit a shortened CDRL1 to accommodate the gp120 N276 glycan, 04_A06 lacks such a deletion, highlighting alternative structural solutions for effective CD4bs engagement. In addition to 04_A06, we identified and structurally characterized 01_D03 and 05_B08, two phylogenetically distinct VRC01-class bnAbs also isolated from donor EN02. Like 04_A06, 01_D03 contacts the adjacent protomer, likely through its FWRH1 and FWRH3 insertions. The four-residue DASG FWRH3 insertion is situated in the same position as a WDFD insertion identified in the 3BNC60/3BNC117 bnAb family, which arose in a different individual^11,45^. Analysis of clonal relatives of 3BNC60/3BNC117 revealed a correlation between the presence of this insertion and potent neutralizing activity, and its removal from 3BNC60 reduced its ability to neutralize diverse viruses^45^, further highlighting the potential importance of antibody insertions for antiviral activity.

Effective HIV-1 prevention approaches will be required to end the pandemic. Although promising concepts for active vaccination strategies are being explored^4^, an effective vaccine remains elusive. Passive immunization with bnAbs represents an alternative, comparable to the use of long-acting antiretrovirals^13,46^. The AMP trials revealed that prevention strategies depend on bnAbs with both high potency and breadth^13,38^. Modeling indicates that a single administration of 04_A06LS at a standard dose and under standard half-life assumptions could provide similar PE as triple bnAb combinations under early clinical investigation^38,47^. However, our in silico predictions are based on PK data from hFcRn mice that may not accurately reflect human PKs. Among groups at risk of HIV-1 infection, newborns remain particularly vulnerable. Although antiretrovirals are authorized for postnatal prophylaxis, drug tolerance and dose finding in children remains challenging. In such groups, long-acting bnAbs represent a safe alternative with extended protection provided during breastfeeding and high treatment adherence^48^. In summary, by conducting an extensive single-cell analysis on a cohort of top HIV-1 elite neutralizers, we identified 04_A06, a bnAb to the CD4bs, that achieved high antiviral activity and restriction of viral escape through unique structural features and has promising potential for clinical development.

## Methods

### Study participants and collection of clinical samples

Large blood draw and leukapheresis samples were collected under protocols approved by the Institutional Review Board (IRB) of the University of Cologne (protocols 13-364 and 16-054) and local IRBs. Participants were recruited from private practices and hospitals in Germany (Cologne, Essen and Frankfurt), Cameroon (Yaoundé), Nepal (Kathmandu) and Tanzania (Mbeya) and provided written informed consent. Compensation was provided in line with institutional and ethical guidelines to reimburse time and expenses without exerting undue influence. In total, serum samples from 2,354 participants were screened for HIV-1 neutralizing activity to identify elite neutralizers. Thirty-two elite neutralizers, representing the top 3.7% of the cohort, were selected for large blood draw collection and B cell isolation. Biosample collection was conducted irrespective of sex/gender (female: 15; male: 17), which was not a study design criterion. Clinical information was obtained from medical records.

### Cell lines

HEK293T cells (ATCC) were cultured in DMEM (Thermo Fisher) with 10% fetal bovine serum (FBS; Sigma-Aldrich), antibiotic–antimycotic (Thermo Fisher), 1 mM sodium pyruvate (Gibco) and 2 mM l-glutamine (Gibco) at 37 °C with 5% CO_2_. HEK293-6E cells (National Research Council of Canada) were maintained in FreeStyle 293 Expression Medium (Life Technologies) with 0.2% penicillin/streptomycin under constant shaking (90–120 rpm) at 37 °C with 6% CO_2_. TZM-bl cells (NIH AIDS Reagent Program) were cultured in DMEM with 10% FBS, 1 mM sodium pyruvate, 2 mM l-glutamine, 50 µg ml^−1^ gentamicin (Merck) and 25 mM HEPES (Millipore) at 37 °C with 5% CO_2_. All cell lines are of female origin and were not further authenticated.

### Mouse models

NRG mice were obtained from The Jackson Laboratory and bred under specific pathogen-free conditions at the University of Cologne’s Decentralized Animal Husbandry Network. Animals were maintained on a 12-h light/12-h dark cycle at 20–22 °C and 30–60% humidity, with ssniff 1124 feed for breeding and ssniff 1543 feed for experiments. Humanized mice were generated as described previously^50^ with modifications. CD34^+^ hematopoietic stem cells were isolated from cord blood or placental tissue using CD34 microbeads (Miltenyi Biotec) under protocols approved by the University of Cologne IRB (16-110) or the Ethics Committee of the Medical Association of North Rhine (2018382), with informed donor consent. Within 5 days after birth, NRG mice received sublethal irradiation and intrahepatic injection of CD34^+^ cells 4–6 h later. Humanization was confirmed 12 weeks after engraftment by FACS analysis of peripheral blood mononuclear cells (PBMCs)^50^. Complementary experiments used already humanized NOD-*Prkdc*^scid^-*Il2rg*^Tm1^/Rj (NXG-HIS) mice from Janvier Labs. All procedures were approved by LANUV (North Rhine-Westphalia).

### Isolation of PBMCs and plasma

PBMCs were isolated from large blood draw samples by density gradient centrifugation using Histopaque (Sigma-Aldrich) and Leucosep tubes (Greiner Bio-One) following the manufacturer’s protocol. PBMCs were cryopreserved at −150 °C in 90% FBS with 10% DMSO, and plasma was stored at −80 °C.

### Isolation of single anti-HIV-1-reactive B cells

Single antigen-reactive B cells were isolated as described previously^14^. CD19^+^ B cells were enriched from PBMCs using CD19 microbeads (Miltenyi Biotec) and stained with DAPI (Thermo Fisher), anti-human CD20–Alexa Fluor 700 (clone 2H7, BD Bioscience, 560631, RRID: AB_2687799), anti-human IgG–APC (clone G18-145, BD Bioscience, 550931, RRID: AB_2738854) and either GFP-labeled BG505_SOSIP.664_ or biotinylated YU2gp_140_ HIV-1 Env bait (15 µg ml^−1^). DAPI^−^CD20^+^ Env-reactive IgG^+^ single cells were sorted into 96-well plates using a FACSAria Fusion (BD). Wells contained 4 µl of buffer (0.5× PBS, 0.5 U µl^−1^ RNasin (Promega), 0.5 U µl^−1^ RNaseOut (Thermo Fisher) and 10 mM DTT (Thermo Fisher)) and were cryopreserved at −80 °C immediately after sorting.

### B cell receptor amplification and sequence analysis

cDNA generation and amplification of antibody heavy and light chain genes from sorted single cells were performed as described previously^14^. For reverse transcription, HIV-1-reactive B cells were incubated with 7 µl of a random hexamer primer mix (Thermo Fisher Scientific, Promega) at 65 °C for 1 min, followed by the addition of Superscript IV RT reagents (Thermo Fisher Scientific) and incubation at 42 °C for 10 min, 25 °C for 10 min, 50 °C for 10 min and 94 °C for 5 min. Heavy and light chains were amplified from cDNA using seminested single-cell PCR with optimized V gene-specific primers^14^ and Platinum Taq or Platinum Taq Green Hot Start polymerase (Thermo Fisher Scientific). Amplicons were verified by agarose gel electrophoresis and subjected to Sanger sequencing. Sequences with a mean Phred score of ≥28 and length of ≥240 nucleotides were retained. Variable regions (FWR1 to J gene end) were annotated with IgBlast^51^ according to IMGT^18^, with low-quality base calls (Phred of <16) masked; sequences with >15 masked bases, stop codons or frameshifts were excluded. Clonality analyses were performed per participant, grouping productive heavy chains by V_H_/J_H_ gene pairs and CDRH3 similarity (≥75% amino acid identity relative to the shortest CDRH3) using Levenshtein distance. Clonal assignment was repeated across 100 randomized input orders, with the solution yielding the fewest unassigned sequences retained. All clones were cross-validated based on shared mutations and paired light chain information.

### Cloning and production of mAbs

Heavy and light chain variable regions of selected antibodies were cloned into expression vectors using sequence- and ligation-independent cloning as described previously^14^. First-round PCR products were amplified with Q5 Hot Start High Fidelity DNA Polymerase (New England Biolabs) using primers containing adaptor sequences homologous to antibody expression vectors (IgG1, IgL and IgK). Multiplexed forward primers encoded the complete native leader sequence of all heavy and light chain V genes, whereas reverse primers targeted conserved motifs at the 5′ end of immunoglobulin constant regions^14^. PCR products were purified using 96-well silica membranes. Modified antibody variants with amino acid insertions or deletions were generated from synthesized heavy chain variable region DNA fragments (Integrated DNA Technologies). Purified PCR products or synthesized fragments were cloned into linearized vectors with T4 DNA polymerase (New England Biolabs) and transformed into *Escherichia coli* DH5α. Correct insertion was verified by Sanger sequencing, and positive colonies were expanded.

Antibody production was performed in both high- and low-throughput formats as previously described^14^. High-throughput expression enabled rapid screening of neutralizing antibodies from 32 HIV-1 elite neutralizers, while confirmed hits were produced in larger scale using the low-throughput method. mAbs were expressed in HEK293-6E suspension cells or HEK293T adherent cells by co-transfection of heavy and light chain vectors using branched PEI (25 kDa, Sigma-Aldrich) or TurboFect (Thermo Fisher). HEK293-6E cells were cultured in FreeStyle 293 medium (Thermo Fisher) with 0.2% penicillin/streptomycin at 37 °C and 6% CO_2_ with shaking at 90–120 rpm for 5–7 days, whereas HEK293T cells were cultured in DMEM (Thermo Fisher) supplemented with 10% FBS (Sigma-Aldrich), antibiotic–antimycotic, 1 mM sodium pyruvate (Gibco) and 2 mM l-glutamine (Gibco) for 4 days.

For cryo-EM studies, heavy chain variable regions of antibodies 04_A06, 05_B08 and 01_D03 were subcloned into a mammalian Fab expression vector containing a C-terminal hexahistidine tag and coexpressed with corresponding light chains in Expi293F cells (Thermo Fisher). Fabs were purified from culture supernatants by Ni-NTA affinity chromatography (GE Life Sciences), buffer exchanged into TBS (20 mM Tris (pH 8.0) and 150 mM NaCl) using Amicon 10-kDa concentrators (Millipore) and further purified by size-exclusion chromatography (SEC) on a Superdex-200 16/60 column equilibrated in TBS. Fractions corresponding to the Fab peak were pooled, concentrated with Amicon 10-kDa concentrators and stored at 4 °C.

### Expression and purification of BG505_SOSIP_ trimer

BG505_SOSIP.664_ for cryo-EM was expressed by transient co-transfection with a furin-encoding plasmid in Expi293F cells (Thermo Fisher) as previously described^52^. Proteins were purified from supernatants by 2G12 (04_A06/PGDM1400 complex) or PGT145 (01_D03, 05_B08 and unbound structure) immunoaffinity chromatography, dialyzed into TBS and concentrated. SEC was performed on a Superose-6 Increase column (PGT145 preps) or sequentially on a Superdex-200 16/60 and Superose-6 Increase 10/300 GL column (2G12 preps) in TBS. Fractions were stored individually at 4 °C.

### Protein G-based antibody purification

Supernatants from HEK293-6E cells transiently transfected with heavy and light chain vectors were collected by centrifugation, filtered (PES, Cytiva) and incubated overnight at 4 °C with Protein G Sepharose beads (GE Life Sciences). Bound IgGs were washed with PBS, eluted with 0.1 M glycine (pH 3) and neutralized with 1 M Tris (pH 8). Buffer exchange to PBS and concentration were performed using 30-kDa Amicon spin membranes (Millipore). Antibodies were sterile filtered (0.22 µm, Millipore), stored at 4 °C and quantified by UV/Vis spectroscopy (Nanodrop, A280).

### Purification of serum IgGs

Serum was diluted 1:1 in DPBS (Gibco, Thermo Fisher) and IgG purified by three passes over a 1.5-ml protein G agarose column (Pierce, Thermo Fisher). After washing with DPBS, bound IgG was eluted with glycine-HCl (pH 2.7) and neutralized with 1 M Tris-HCl (pH 8.0). Buffer exchange to PBS and IgG concentration were performed using 10-kDa molecular-weight-cutoff filters (Thermo Fisher).

### Determination of antibody concentrations by human IgG capture ELISA

Human IgG capture ELISA was used to quantify antibody concentrations in unpurified supernatants from transfected HEK293T or HEK293-6E cells as described previously^14^ with minor modifications. ELISA plates (Greiner Bio-One) were coated with 2.5 µg ml^−1^ polyclonal goat anti-human IgG in PBS (45 min at 37 °C or overnight at 4 °C), blocked with 5% nonfat milk in PBS for 60 min at room temperature and incubated with cell supernatants (starting dilution of 1:20) or serially diluted human myeloma IgG1κ standard (Sigma-Aldrich, I5154; 4 µg ml^−1^). Bound antibodies were detected with anti-human IgG-HRP (Jackson ImmunoResearch, 109-035-098, RRID: AB_237586; 1:2,500 in blocking buffer). Plates were developed with ABTS substrate (Thermo Fisher, 002024), and absorbance was read at 415/695 nm (Tecan). Antibody concentrations were calculated relative to the IgG1 standard.

### HIV-1 pseudovirus production

HIV-1 pseudoviruses were generated in HEK293T cells by co-transfection with pSG3∆*env* and corresponding Env plasmids as previously described^53^. Donor- and mouse-derived Env plasmids were synthesized by Twist Bioscience.

### Neutralization assay

Neutralization assays were performed to determine IC_50_/IC_80_ values of purified mAbs, purified serum IgGs and unpurified HEK293T supernatants as described previously with minor modifications^53^. For donor screening, isolated IgGs were tested against each virus at 300 µg ml^−1^ in duplicate wells. mAbs, IgGs or supernatants were incubated with pseudoviruses for 1 h at 37 °C before the addition of 1 × 10^4^ TZM-bl cells per well. After 48 h at 37 °C and 5% CO_2_, luciferase activity was measured, background relative light units were subtracted, and percent neutralization was calculated. Unpurified IgGs from supernatants were tested at 2.5 µg ml^−1^, with concentrations determined by IgG capture ELISA. For IC_50_/IC_80_ determination, mAbs were serially diluted from 10, 25 or 50 µg ml^−1^ and tested in duplicate; values were calculated in GraphPad Prism as the concentration causing 50% or 80% inhibition relative to virus-only controls. Reference antibodies were validated against the global HIV-1 pseudovirus panel, and IC_50_/IC_80_ values were compared with CATNAP^49^ data. Only antibodies with less than threefold deviation from reference values were included in further analyses.

### Antibody epitope prediction using neutralization fingerprinting

Epitope prediction of serum IgG neutralizing activity was performed computationally as previously described^54^. Neutralization was measured in TZM-bl assays against a 20-virus f61 fingerprinting panel^54^. Polyclonal profiles were deconvoluted into contributions from ten epitope-specific clusters, defined by broadly neutralizing reference antibody fingerprints^55^. Epitope prevalence was estimated by least-squares fitting, generating ten scores (0–1, summing to 1). Scores of ≥0.25 were considered positive, allowing the identification of up to four dominant specificities per serum sample.

### HIV-1 Env ELISAs

High-binding ELISA plates (Greiner Bio-One) were coated with HIV-1 Env protein (4 µg ml^−1^), blocked with 2% bovine serum albumin/0.05% Tween-20 in PBS for 1 h at 37 °C and incubated with serial 1:4 dilutions of mAbs starting at 10 µg ml^−1^. Bound antibodies were detected with anti-human IgG-HRP (Jackson ImmunoResearch, 109-035-098, RRID: AB_237586; 1:1,000 in 2% bovine serum albumin/PBS) for 1 h at room temperature. Plates were washed with PBS/0.05% Tween-20 and developed with ABTS (Thermo Fisher, 002024), and absorbance was measured at 415/695 nm (Tecan). All samples were tested in duplicate.

### Competition ELISAs

Selected mAbs were biotinylated using an EZ-Link Sulfo-NHS-Biotin kit (Thermo Fisher) and buffer exchanged into PBS with Amicon 10-kDa filters (Millipore). High-binding ELISA plates (Greiner Bio-One) were coated with anti-6×His (Abcam, ab9108, RRID: AB_307016; 2 µg ml^−1^) overnight at 4 °C, blocked with 3% bovine serum albumin/PBS for 1 h at 37 °C and incubated with BG505_SOSIP.664_–His (2 µg ml^−1^, 1 h at room temperature). Competing antibodies were added at 32 µg ml^−1^ and serially diluted 1:3, followed by the addition of 0.5 µg ml^−1^ biotinylated test antibodies (1 h at room temperature). Detection was performed with peroxidase–streptavidin (Jackson ImmunoResearch; 1:5,000 in PBS/1% bovine serum albumin/0.05% Tween-20). Plates were washed with PBS/0.05% Tween-20 between steps, developed with ABTS (Thermo Fisher, 002024) and read at 415/695 nm (Tecan).

### Autoreactivity evaluations in HEp-2 cell assays

HEp-2 cell autoreactivity was assessed with a NOVA Lite Hep-2 ANA kit (Inova Diagnostics) following the manufacturer’s protocol. mAbs (100 μg ml^−1^ in PBS) were applied, and images were acquired using a Leica DMI 6000 B fluorescence microscope (3-s exposure, 100% intensity, gain of 10).

### Cryo-EM sample preparation

The 04_A06–PGDM1400–BG505 complex was assembled at a 3.6:1.2:1 molar ratio (Fab:Fab:trimer), incubated overnight at room temperature, purified on a Superose-6 Increase 10/300 GL column and concentrated to ~2.5 mg ml^−1^ in TBS (Amicon 10-kDa, Millipore) 1 day before vitrification. The 01_D03–BG505 and 05_B08–BG505 complexes were prepared at a 3.6:1 molar ratio (Fab:trimer), incubated overnight in TBS at room temperature and concentrated to ~4.2 mg ml^−1^ and ~4.4 mg ml^−1^, respectively, without further SEC. Unbound BG505 was concentrated to ~4.0 mg ml^−1^.

Octyl-maltoside fluorinated solution was added to each sample for a final concentration of 0.02% (wt/vol) immediately preceding the addition of 3 µl to a Quantifoil R1.2/1.3 Cu 300-mesh grid (04_A06–PGDM1400 complex) or a Quantifoil R1.2/1.3 Holey Carbon Film 300-mesh gold grid (01_D03 and 05_B08 complexes and unbound BG505; Electron Microscopy Services) that had been glow discharged for 1 min at 20 mA using a PELCO easiGLOW (Ted Pella). Grids were blotted for 3 s with Whatman No. 1 filter paper and plunge-frozen in liquid ethane using a Mark IV Vitrobot (Thermo Fisher) operating at room temperature and 100% humidity.

### Cryo-EM data collection and processing

Data were collected on a 300-keV Titan Krios transmission electron microscope (Thermo Fisher Scientific) equipped with a GIF Quantum energy filter and a K3 6,000 × 4,000 direct electron detector (Gatan) operating in counting mode. Data collection was performed using SerialEM v4.0.13 (04_A06 and unbound BG505) or v4.1.0beta^56^ (01_D03 and 05_B08) at a nominal magnification of ×105,000 (super-resolution = 0.416 Å per pixel) and a defocus range of –1.0 to –3.0 µm. Movies were recorded using a 3 × 3 beam image shift pattern with one (04_A06/PGDM1400 dataset), two (01_D03 and 05_B08 datasets) or three shots (unbound BG505 dataset) per hole. For the 04_A06 and 01_D03 datasets, motion correction, contrast transfer function estimation, particle picking and binned particle extraction were performed using cryoSPARC Live (v3 and v4, respectively) before processing in cryoSPARC^56^. For the 05_B08 and unbound BG505 datasets, all processing was performed in cryoSPARCv4. Particles were picked using blob picker or Topaz^57^ and extracted from micrographs. For the 04_A06–PGDM1400–BG505 dataset, a model was built into the map containing PGDM1400, and after verifying that this model (minus PGDM1400 and the N160 glycans) fit well into the C3 symmetric map that lacked PGDM1400, particle subtraction and local refinement (with applied C3 symmetry) were performed to obtain a higher-resolution view of the 04_A06–BG505 interface. To create a mask for particle subtraction, the ‘molmap’ command in ChimeraX was applied to a model of the PGDM1400 Fab and gp120 N160 glycans that was built into the EM density. The mask was imported into cryoSPARC^56^, and a soft padding was applied (threshold = 0.1; soft padding width = 10 voxels). A mask for local refinement was similarly created using a model of BG505 and the 04_A06 V_H_V_L_ (threshold = 0.05, dilation radius = 5; soft padding width = 10).

### Structure modeling and refinement

The following coordinates were docked into the corresponding densities of the EM maps using ChimeraX^58^ to generate starting models: BG505 (PDB: 6UDJ), PGDM1400 (PDB: 4RQQ) and the 04_A06 Fab crystal structure (PDB: 8UKI; this study). Sequence-corrected models for the 01_D03 and 05_B08 V_H_V_L_ domains were built into the corresponding densities in Coot^59^ using the 04_A06 Fab crystal structure as a starting model. Models were refined through iterative rounds of Phenix real space refine and Coot. N-Glycans were built using tools in Coot^59^ and verified as ‘OK’ by Privateer^60^. Modeling of side chains should be considered approximate, owing to the intermediate resolution of the EM structures. Antibody residues were numbered according to Kabat.

### X-ray crystallography

Crystallization screens for the 04_A06 Fab were performed using sitting drop vapor diffusion at room temperature by mixing 0.2 µl of Fab (4.1 mg ml^−1^) with 0.2 µl of reservoir solution (Hampton Research) using a TTP Labtech Mosquito automatic microliter pipetting robot. 04_A06 Fab crystals were obtained in 8% (vol/vol) Tacsimate (pH 7.0) and 20% (wt/vol) polyethylene glycol 3350. Crystals were looped and cryopreserved in reservoir solution supplemented stepwise with 5–20% glycerol and cryopreserved in liquid nitrogen. A 1.75-Å structure of 04_A06 Fab was solved using a dataset collected at 100 K and a 1-Å wavelength on Beamline 12-2 at the Stanford Synchrotron Radiation Lightsource with an Eiger X 16M (Dectris) detector, which was indexed and integrated with iMosflm v7.4 and then merged with AIMLESS in the CCP4 software package v7.1.018.

The structure was determined by molecular replacement in Phaser with the coordinates of the VRC01 Fab (PDB: 3NGB), using C_H_–C_L_ and V_H_–V_L_ (with truncated CDR loops) as separate search models. Coordinates were refined using PHENIX v1.20.1-4487129 with individual B factors and TLS restraints^61^. Manual rebuilding was performed iteratively with Coot v0.9.8.8131 (ref. ^62^). A total of 99.1% of residues were in the favored regions of the Ramachandran plot, and 0.9% were in the allowed region (Supplementary Table 9).

### Structural analyses

Figures were prepared using UCSF ChimeraX^34^ and PyMOL (Schrödinger). Buried surface area was calculated using PDBePISA v1.52 (ref. ^63^) with a 1.4-Å probe. The r.m.s.d. values were calculated in PyMOL (Schrödinger), and electrostatic surfaces were calculated in UCSF ChimeraX^34^. Owing to the intermediate resolution and minor differences in modeling of identical copies of chains within a trimer, a general cutoff of ≤6.0 Å was used to define potential interactions.

### Generation of mutant HIV-1_YU2_ and HIV-1_BG505_ pseudovirus mutants

Point mutations were introduced into HIV-1_YU2_ or HIV-1_BG505_ gp160 plasmids using a Q5 Site-Directed Mutagenesis kit (New England Biolabs) per the manufacturer’s protocol, and pseudovirus mutants were generated as described above.

### DMS

A lentivirus DMS platform was applied to quantitatively assess the impact of mutations and combinations thereof on the functionality of the HIV-1 Env protein and resistance to neutralization by antibodies as previously described^36,37^. For the present investigation, we deployed mutant libraries of the HIV-1 virus strain BF520 to explore antibody resistance patterns. In brief, two distinct BF520 Env mutant lentivirus libraries were used, each containing around 40,000 mutants with an average of approximately 2.5 nonsynonymous mutations per mutant. For antibody selection, VSV-G pseudotyped neutralization standard viruses were added to virus pools to make up 0.5–1% of the total. Each selection condition involved incubating 1 million infectious units from one of the mutant libraries containing the pseudotyped standard with various dilutions of antibodies, spanning concentrations from IC_90_ to IC_99.9_, along with a mock incubation for control purposes. Following a 1-h incubation period, the mixtures were used to infect 1 million TZM-bl cells per well in a six-well plate, with the addition of 100 µg ml^−1^ DEAE dextran to enhance infection efficiency. Twelve hours after infection, the unintegrated lentivirus genomes from each condition were extracted and processed for sequencing as previously described^37^.

### DMS data analysis

The dms-vep-pipeline-3 (version 3.2.3) was applied to analyze generated DMS data (https://github.com/dms-vep/dms-vep-pipeline-3), and the full analysis including CSV files with numerical measurements and HTML renderings of key analyses was deposited in a GitHub repository (https://dms-vep.github.io/HIV_Envelope_BF520_DMS_04-A06). Analysis of PacBio and Illumina sequencing data, as well as modeling of mutation effects on HIV Env function and escape from neutralizing antibodies, were conducted as previously described^36,37^. In brief, to model antibody escape, we estimated the fraction of each mutant that was not neutralized in each antibody selection by comparing the counts of each mutant to those of the non-neutralized standard viruses under both antibody and mock incubation conditions. To quantify the effects of each individual mutation on escape from each antibody, the software package polyclonal version 6.6 (https://jbloomlab.github.io/polyclonal) was applied. The logo plots (Extended Data Fig. 8) display the modeled effects of each individual mutation on escape from each antibody, where the height of the letter of the amino acid represents the magnitude of the measured effect on escape.

### Production of recombinant HIV-1 virus

Replication-competent HIV-1 (YU2 *env* in the NL4-3 backbone) was generated by transfecting HEK293T cells with FuGENE 6 (Promega). Viral supernatants were collected 48–72 h after transfection and stored at −80 °C.

### Viral outgrowth of replication-competent isolates

CD4^+^ T cells were isolated from PBMCs of PLWH using a CD4^+^ T Cell Isolation kit (Miltenyi Biotec) and cocultured with irradiated (50 Gy) PBMCs from healthy donors in T cell medium (RPMI 1640, 300 mg l^−1^ l-glutamine, 10% FBS and 1% penicillin/streptomycin). Cultures were stimulated with 1 µg ml^−1^ PHA-M (Sigma-Aldrich) and 100 U ml^−1^ interleukin-2 (IL-2; Miltenyi Biotec). After 1 day, the medium was replaced with IL-2 (100 U ml^−1^) and polybrene (5 µg ml^−1^), and CD8^+^-depleted, PHA/IL-2-stimulated donor PBMCs were added. Cultures were supplemented weekly with additional CD8^+^-depleted donor PBMCs. Supernatants were monitored for p24 antigen (Architect HIV Ag/Ab Combo, Abbott) and were collected after detection, followed by storage at −80 to −150 °C.

### Infection of humanized mice and viral load measurements

Humanized NRG or NXG-HIS mice were challenged intraperitoneally with replication-competent HIV-1_YU2_. At 21–24 days after challenge, only mice with viral loads of >7,870 copies per ml (NRG, day −1) or >2,000 copies per ml (NXG-HIS, day −2) were included (NRG: *n* = 36, 20 male/16 female, 19.3–40.7 weeks; NXG-HIS: *n* = 28, all female, 32–37 weeks). Mice were randomized into treatment or control groups based on viral load, age and time since humanization. Treatments consisted of subcutaneous injections of sterile mAbs in PBS (1 mg loading dose, then 0.5 mg every 3–4 days).

Viral RNA was extracted from EDTA plasma using a MinElute Virus Spin kit with DNase I on a Qiacube (Qiagen). Viral loads were quantified by quantitative PCR with reverse transcription (QuantStudio 5, Thermo Fisher) using *pol*-specific primers and probe^64^ with a TaqMan RNA-to-*C*_t_ 1-Step kit. Standards were prepared from heat-inactivated HIV-1_YU2_ supernatants (SupT1-R5 cells) and quantified with a cobas 6800 HIV-1 kit (Roche). Limits of detection were 784 copies per ml (NRG) and 451 copies per ml (NXG-HIS). For log_10_ calculations, viral loads below detection were set to 783 or 451 copies per ml, respectively.

### SGS of plasma HIV-1 *env* from humanized mice

SGS was performed as previously described^65^. Viral RNA was extracted from EDTA plasma using a MinElute Virus Spin kit with DNase I on a Qiacube (Qiagen). Reverse transcription was performed with primer YB383 5′-TTTTTTTTTTTTTTTTTTTTTTTTRAAGCAC-3′ (ref. ^66^) and SuperScript IV (Thermo Fisher), followed by RNase H treatment (0.25 U µl^−1^, 37 °C, 20 min). *env* cDNA was serially diluted and amplified by nested PCR using Platinum Taq Green Hot Start (Thermo Fisher) with HIV-1_YU2-NL4-3_-specific primers. The first PCR used YB383 and YB50 (5′-GGCTTAGGCATCTCCTATGGCAGGAAGAA-3′), and the second PCR used YB49 (5′-TAGAAAGAGCAGAAGACAGTGGCAATGA-3′) and YB52 (5′- GGTGTGTAGTTCTGCCAATCAGGGAAGWAGCCTTGTG-3′). Cycling conditions were 94 °C for 2 min, 35 or 45 cycles of 94 °C for 30 s, 55 °C for 30 s and 72 °C for 4 min, with a final extension at 72 °C for 15 min. PCRs with <30% efficiency were purified using a NucleoSpin 96 PCR Clean-Up kit (Macherey-Nagel).

### Illumina dye sequencing of humanized mouse SGS-derived *env* amplicons

SGS-derived *env* amplicons were purified (NucleoSpin 96 PCR Clean-up kit, Macherey-Nagel) and sequenced as 2 × 150 base pair libraries on a NovaSeq at the Cologne Center for Genomics.

### Determination of antibody PKs in vivo

The half-lives of wild-type antibodies (04_A06, 10-1074 and 3BNC117) were determined in human FcRn transgenic mice (B6.Cg-*Fcgrt*^tm1Dcr^*Prkdc*^scid^Tg(FCGRT)32Dcr/DcrJ, Jackson Laboratory; *n* = 12, all female) following intravenous injection of 0.5 mg of purified antibody in PBS. Serum/plasma IgG concentrations were measured by ELISA with minor modifications^50^. Plates (Corning) were coated with anti-human IgG (2.5 µg ml^−1^, Jackson ImmunoResearch), blocked with 2% bovine serum albumin/1 µM EDTA/0.1% Tween-20 and incubated with serial dilutions of human IgG1κ standard (Sigma-Aldrich) or samples, followed by HRP-conjugated anti-human IgG (1:1,000). Plates were developed with ABTS and read at 415 nm (Tecan), with PBS-Tween washes between steps. Baseline samples confirmed the absence of human IgG before injection.

For PK analysis of 04_A06LS and sotrovimab, FcRn mice (*n* = 5, all female) received a single 5 mg per kg (body weight) intravenous bolus (1 mg ml^−1^ in TA vehicle (histidine 20 mM, NaCl 150 mM, pH 6), 5 ml per kg). Blood was collected at 15 time points up to 63 days after treatment via tail vein puncture, clotted 30–45 min at room temperature and centrifuged (5 min, 2–8 °C, 5,500*g*), and the serum was stored at −20 °C. mAb concentrations were measured by MSD electrochemiluminescence assay using anti-LS capture and anti-huCH2-sulfotag detection. PK parameters were calculated in WinNonlin (8.4.0.6172, Certara) using nominal dose/times, standard noncompartmental analysis and linear up/log down rules. Sotrovimab served as a comparator.

### Determination of the predicted PE and GeoMean PT_80_

The predicted PE and PT_80_ were determined following previously described methodology^38^. Calculations were conducted under the scenario of administration of three bnAb infusions every 8 weeks. For each model, the GeoMean PT_80_ was computed for 1,000 simulated participants, akin to those in the AMP trial, against panels of 31 clade C AMP placebo viruses, 86 clade C AMP VRC01 breakthrough viruses and 68 clade B AMP placebo viruses. For each simulated participant, the PT_80_ was calculated as the predicted steady-state serum concentration of 04_A06 divided by the GeoMean of the in vitro IC_80_ for the viruses in the AMP trial. These calculations assumed that 04_A06 displays the PK profile of either 3BNC117 or 10-1074. PE was estimated by assuming that the PT_80_ needed for a specific level of PE is twice as high as that found in nonhuman primate studies according to a meta-analysis, presenting a more conservative approach than that seen in the AMP trials^38^.

### Sequence annotation and clone assignment

Sequences were filtered for a minimum Phred score of 28 and ≥240 nucleotides, annotated with IgBLAST, and trimmed to the variable region (FWR1–J gene)^51^. Bases with a Phred score of <16 were masked, and sequences with >15 masked bases, stop codons or frameshifts were excluded. For each donor, productive heavy chain sequences were grouped by V_H_/J_H_ gene pairs, and CDRH3 similarity was assessed by pairwise Levenshtein distance. Clones were defined by ≥75% CDRH3 identity relative to the shortest sequence. Clonal assignment was repeated over 100 randomizations, and the solution with the fewest unassigned sequences was used. All clones were cross-validated by shared mutations and paired light chain data.

### Inference of the phylogenetic tree

Phylogenetic relationships of clones 7, 9 and 1 (Fig. 2) were inferred by aligning nucleotide sequences with Clustal Omega^67^. Heavy and light chain sequences were concatenated to form extended sequences, and germline sequences with masked CDR3 regions were included. Trees were reconstructed in RAxML using the GTRGAMMA model and rooted at the germline. Branch lengths represent mutations per nucleotide, with the initial edge from root to Most Recent Common Ancestor reflecting only V and J gene mutations, as germline CDR3 is unavailable.

### Determination of intra- and interdonor bnAb similarities

The similarity metric in Fig. 2 is the fraction of shared V_H_ gene mutations relative to the *IGHV1-2* germline. All V genes from donor bnAbs and the germline were aligned with Clustal Omega^67^. Mutations were counted relative to the germline, treating gaps as nucleotides. Pairwise similarity was defined as the fraction of germline mutations shared between two sequences. The similarity measure was then defined as follows:

$${\rm{Fraction}}\,{\rm{common}}\,{\rm{mutant}}\,{\rm{V}}_{\rm{H}}=\frac{N_{{\rm{common}}}\,{{\rm{mutant}}}}{(N_{{\rm{mutant}}}\,{{\rm{sequence}}}\,1)(N_{{\rm{mutant}}}\,{{\rm{sequence}}}\,2)}$$

### Use of large language models

ChatGPT (v.4 and v.5) was used for general editorial tasks, including proof-reading, grammar correction and text summarization only. Scientific content and conclusions are the work of the authors.

### Statistics and reproducibility

Flow cytometry analyses and quantification were performed using FlowJo10 software. Statistical tests and analyses were performed with GraphPad Prism (v7 and v8), Python (v3.6.8), R (v4.0.0) and Mircosoft Excel for Mac (v14.7.3 and 16.4.8). CDRH3 lengths, V gene usage and germline identity distributions for clonal sequences were assessed for all input sequences without further collapsing. No statistical method was used to predetermine sample size. No data were excluded from analyses. The experiments were not randomized. The investigators were not blinded to allocation during experiments, the condition of the experiments or outcome assessment. No statistical methods were used to predetermine sample sizes, but our sample sizes are similar to those reported in previous publications^5–9^.

### Inclusion and ethics

Research has been conducted and authorship has been determined in alignment with the Global Code of Conduct for Research in Resource-Poor Settings.

### Reporting summary

Further information on research design is available in the Nature Portfolio Reporting Summary linked to this article.

## Online content

Any methods, additional references, Nature Portfolio reporting summaries, source data, extended data, supplementary information, acknowledgements, peer review information; details of author contributions and competing interests; and statements of data and code availability are available at 10.1038/s41590-025-02286-5.

## Supplementary information

Reporting Summary

Supplementary Tables 1–12 Table 1. Demographic and clinical characteristics of analyzed HIV-1 elite neutralizers. Table 2. Neutralizing activity of isolated mAbs to an HIV-1 pseudovirus screening panel. Table 3. Isolated HIV-1-reactive B cell clones and sequences isolated from individual EN02. Table 4. Neutralizing activity against a global pseudovirus panel and binding activity of isolated mAbs from EN02. Table 5. Neutralizing activity of bNAbs 04_A06, 01_D03 and 05_B08 to the 119 multiclade pseudovirus panel. Table 6. Neutralizing activity of bNAbs 04_A06, 01_D03 and 05_B08 to the 208 multiclade pseudovirus panel. Table 7. Neutralizing activity of bNAb 04_A06 to the subtype C pseudovirus panel. Table 8. Neutralizing activity of bNAbs 04_A06 and VRC07523-LS to replication-competent viral isolates. Table 9. Cryo-EM data collection and refinement statistics. Table 10. SGS-derived *env* sequences from monotherapy groups of in vivo experiments. Table 11. SGS-derived *env* sequences from combination therapy groups of in vivo experiments. Table 12. Neutralizing activity of bNAb 04_A06 to the pseudoviruses generated from the AMP trials.

## Data Availability

Nucleotide sequences of isolated V_H_1-2-encoded bnAbs to the CD4bs have been deposited at GenBank under accession codes PX149247–PX149310. The next-generation sequencing B cell repertoire data analyzed in this study have been deposited in the Sequence Read Archive under accession codes SAMN29624595–SAMN29624713 and the BioProject database under accession code PRJNA857338. Cryo-EM maps and models have been deposited in the Electron Microscopy Data Bank and PDB under accession codes EMD-46649 and 9D8V (unbound BG505), EMD-42363 and 8ULR (05_B08–BG505v2), EMD-42364 and 8ULS (01_D03–BG505), EMD-42365 and 8ULT (04_A06–BG505) and EMD-42366 and 8ULU (04_A06–PGDM1400–BG505). Coordinates for the 04_A06 Fab crystal structure have been deposited to the PDB under accession code 8UKI. Aggregated clinical data are available upon request to the corresponding author (F.K.) provided that there is no reasonable risk of deanonymizing study participants and/or may require a Material Transfer Agreement. Individual donor data cannot be shared due to privacy restrictions. Requests will be responded to within 2 weeks.

## Code availability

All computer code and data for the DMS analysis are publicly available on GitHub (https://github.com/dms-vep/HIV_Envelope_BF520_DMS_04-A06). Code implementation for in silico modeling of the predicted PE is described in Gilbert et al.^68^ and is publicly available at http://faculty.washington.edu/peterg/programs.html?.
