# Supplementary material for: Profiling of HIV-1 elite neutralizer cohort reveals a CD4bs bnAb for HIV-1 prevention and therapy
Source: Nat Immunol. 2025 Oct 6;26(11):2016–29. doi: 10.1038/s41590-025-02286-5 (PMC12571865; doi:10.1038/s41590-025-02286-5)
Supplement: Supplementary file 1 [file 41590_2025_2286_MOESM1_ESM.pdf]

nature portfolio

Corresponding author(s): Florian Klein

Last updated by author(s): Aug 19, 2025

Reporting Summary

Nature Portfolio wishes to improve the reproducibility of the work that we publish. This form provides structure for consistency and transparency in reporting. For further information on Nature Portfolio policies, see our Editorial Policies and the Editorial Policy Checklist.

Statistics

For all statistical analyses, confirm that the following items are present in the figure legend, table legend, main text, or Methods section.

n/a	Confirmed	
☐	☒	The exact sample size (*n*) for each experimental group/condition, given as a discrete number and unit of measurement
☐	☒	A statement on whether measurements were taken from distinct samples or whether the same sample was measured repeatedly
☐	☒	The statistical test(s) used AND whether they are one- or two-sided<br>*Only common tests should be described solely by name; describe more complex techniques in the Methods section.*
☒	☐	A description of all covariates tested
☒	☐	A description of any assumptions or corrections, such as tests of normality and adjustment for multiple comparisons
☐	☒	A full description of the statistical parameters including central tendency (e.g. means) or other basic estimates (e.g. regression coefficient) AND variation (e.g. standard deviation) or associated estimates of uncertainty (e.g. confidence intervals)
☐	☒	For null hypothesis testing, the test statistic (e.g. *F*, *t*, *r*) with confidence intervals, effect sizes, degrees of freedom and *P* value noted<br>*Give P values as exact values whenever suitable.*
☒	☐	For Bayesian analysis, information on the choice of priors and Markov chain Monte Carlo settings
☒	☐	For hierarchical and complex designs, identification of the appropriate level for tests and full reporting of outcomes
☐	☒	Estimates of effect sizes (e.g. Cohen's *d*, Pearson's *r*), indicating how they were calculated

Our web collection on statistics for biologists contains articles on many of the points above.

Software and code

Policy information about availability of computer code

Data collection	GraphPad Prism V.8: Illustration of data and statistical parameter. Statistical analysis of data.<br>Microsoft Excel V16.16.27 (201012): Illustration of data and statistical parameter. Statistical analysis of data<br>BD FACSDiva 6.1.2: Generation of flow cytometry data.<br>ELISA measurements were performed using Tecan's Sunrise absorbance microplate reader and associated software.<br>Neutralizing activity and luminescence were measures using BertholdTech Tristar2S and associated device software<br>B cell repertoire sequence data was generated by an unbiased template-switch-based NGS approach (Ehrhardt et al., Nature Medicine 2019)<br>Sanger sequencing of antibody v genes were gained by Eurofins genomics.<br>Clinical data for some study individuals was documented in an electronic case report form (eCRF) using the online cohort platform ClinicalSurveys.net hosted by QuestBack, Oslo, Norway on servers of UHC, Cologne, Germany, as part of a software-as-a-service agreement.
Data analysis	Data, quantifications and statistical analyses were performed using GraphPad Prism V.8, Microsoft Excel V16.16.27 (201012), BD FlowJo V.10, and Geneious Prime (v.2020.2.4). Raw read pre-processing of B cell NGS data was performed with an in-house pipeline primarily basedon self-written Python scripts, IgBLAST, Clustal Omega, and the pRESTO toolkit. Raw IGHV and immunoglobulin light chain variable (IGLV) sequences derived from NGS and single B cell sequencing data were annotated to human V, D, and J germline reference sequences using MiXCR software.

For manuscripts utilizing custom algorithms or software that are central to the research but not yet described in published literature, software must be made available to editors and reviewers. We strongly encourage code deposition in a community repository (e.g. GitHub). See the Nature Portfolio guidelines for submitting code & software for further information.

Data

Policy information about availability of data

All manuscripts must include a data availability statement. This statement should provide the following information, where applicable:

- Accession codes, unique identifiers, or web links for publicly available datasets
- A description of any restrictions on data availability
- For clinical datasets or third party data, please ensure that the statement adheres to our policy

Nucleotide sequences of isolated VH1-2-encoded CD4bs bNAbs have been deposited at GenBanku under accession codes PX149247-PX149310. The NGS B cell repertoire data analysed in this study have been deposited in the Sequence Read Archive (SRA) under accession codes SAMN29624595 to SAMN29624713 [https://www.ncbi.nlm.nih.gov/sra?linkname=bioproject_sra_all&from_uid=857338] and the BioProject database under accession code PRJNA857338.Cryo-EM maps and models have been deposited in the Electron Microscopy Data Bank (EMDB) and Protein Data Bank with accession codes: EMD-46649 and PDB ID 9D8V (unbound BG505), EMD-42363 and PDB ID 8ULR (05_B08-BG505v2), EMD-42364 and PDB ID 8ULS (01_D03-BG505), EMD-42365 and PDB ID 8ULT (04_A06-BG505), and EMD-42366 and PDB ID 8ULU (04_A06-PGDM1400-BG505). Coordinates for the 04_A06 Fab crystal structure have been deposited to the Protein Data Bank with accession code 8UKI. Aggregated clinical data are available upon request to the corresponding author (F.K.) provided that there is no reasonable risk of de-anonymizing study participants and/or may require a Material Transfer Agreement (MTA). Individual donor data cannot be shared due to privacy restrictions. Requests will be responded to within 2 weeks.

Research involving human participants, their data, or biological material

Policy information about studies with human participants or human data. See also policy information about sex, gender (identity/presentation), and sexual orientation and race, ethnicity and racism.

Reporting on sex and gender	In this study, we collected data about the sex of study individuals. No data was collected on gender. Therefore, we only use the term "sex" in our manuscript. We present data from 15 female and 17 male study participants. Samples were collected irrespective of sex or gender. Sex or gender was not relevant to the study design.
Reporting on race, ethnicity, or other socially relevant groupings	The origin of samples was determined by the location of sample acquisition.<br>1.) Germany 25%<br>2.) Tanzania 44%<br>3.) Nepal 25%<br>4.) Cameroon 6%
Population characteristics	Population characteristic about sex of the described study individuals are as following:<br>1.) Male sex: Germany 88%; Tanzania 43%; 25% Nepal; 50% Cameroon.<br>2.) Median age: Germany 40 years; Tanzania 39 years; Nepal 38 years; Cameroon 42 years
Recruitment	Patients were recruited from private practices and hospitals in Germany (Cologne, Essen, Frankfurt), Cameroon (Yaoundé), Nepal (Kathmandu), and Tanzania (Mbeya). Recruitment occurred during routine visits to these facilities. Enrollment was unbiased as it was determined by the treating physician without prior selection or self-selection by the patients. However, the physician's choice could introduce potential bias in patient selection. While we recognize this possible source of bias in our recruitment approach, we are confident that it did not influence the study outcomes.
Ethics oversight	Large-blood draw and leukapheresis samples were collected according to protocols reviewed and approved by the Institutional Review Board (IRB) of the University of Cologne (study protocols 13-364 and 16-054) and local IRBs. Compensation was provided in line with institutional and ethical guidelines to reimburse time and expenses without exerting undue influence.

Note that full information on the approval of the study protocol must also be provided in the manuscript.

Field-specific reporting

Please select the one below that is the best fit for your research. If you are not sure, read the appropriate sections before making your selection.

☒ Life sciences ☐ Behavioural & social sciences ☐ Ecological, evolutionary & environmental sciences

For a reference copy of the document with all sections, see nature.com/documents/nr-reporting-summary-flat.pdf

Life sciences study design

All studies must disclose on these points even when the disclosure is negative.

Sample size	No sample-size calculations were relevant for our study.
Data exclusions	For NGS data, reads were initially filtered for a mean Phred score of 25 and read-lengths of at least 250 bp. Consensus sequences (based on UMIs) were excluded, when the corresponding UMI was found less than 3 times. For sequence analysis of single cell-derived v gene sequences, chromatograms were first filtered based on a minimum Phred score of 28 and a length of at least 240 nucleotides. Sequences were then processed using IgBLAST to annotate them, and trimmed to extract only the variable region, extending from FWR1 to the end of

	the J gene. Base calls within the variable region with a Phred score below 16 were masked. Sequences containing over 15 masked nucleotides, stop codons, or frameshift mutations were excluded from subsequent analysis. No other data were excluded from analyses.
Replication	For antibody screening assays, the neutralizing activity of monoclonal antibodies was first assessed without duplicates. Determination of the neutralizing activity of antibodies against other virus panels were conducted in duplicates and the geometric mean was reported.
Randomization	Randomization was not relevant for our study.
Blinding	Researches conducting experiments were aware of the study ID for each individual they handled, but they were blinded to the participants' clinical data.

Reporting for specific materials, systems and methods

We require information from authors about some types of materials, experimental systems and methods used in many studies. Here, indicate whether each material, system or method listed is relevant to your study. If you are not sure if a list item applies to your research, read the appropriate section before selecting a response.

Materials & experimental systems

n/a	Involved in the study	
☐	☒	Antibodies
☐	☒	Eukaryotic cell lines
☒	☐	Palaeontology and archaeology
☐	☒	Animals and other organisms
☐	☒	Clinical data
☒	☐	Dual use research of concern
☒	☐	Plants

Methods

n/a	Involved in the study	
☒	☐	ChIP-seq
☐	☒	Flow cytometry
☒	☐	MRI-based neuroimaging

Antibodies

Antibodies used	Anti-human CD20-AF700 (clone 2H7), BD Bioscience, Cat#560631, RRID: AB_2687799<br>Anti-human IgG-APC (clone G18-145), BD Bioscience, Cat#550931, RRID AB_2738854<br>Peroxidase AffiniPure Goat Anti-human IgG, Fcγ fragment specific, Jackson ImmunoResearch, Cat#109-035-098, RRID: AB_237586<br>IgG1 kappa from human myeloma plasma (Sigma Aldrich, cat. no. I5154)<br>Rabbit anti-6x His tag polyclonal antibody, unconjugated (Abcam Cat# ab9108, RRID:AB307016)<br>Monoclonal anti-HIV-1 bNAb VRC01, NIH Aids Reagent Program Cat#12033; RRID: AB_2491019<br>Monoclonal anti-HIV-1 bNAb N6, NIH Aids Reagent Program Cat#12968<br>Monoclonal anti-HIV-1 bNAb 3BNC117, NIH Aids Reagent Program Cat#12474; RRID: AB_2491033<br>Monocloanl anti-HIV-1 bNAb 1-18, Genbank ID heavy chain: MN867953.1, light chain: MN868009.1
Validation	All antibodies from BD bioscience and Jackson ImmunoResearch were controlled for human reactivity during manufcaturer's quality control used for cell sorting and ELISA experiments. All anti-HIV-1 bNAbs reference antibodies were functionally validated through neutralization assays against the global HIV-1 pseudovirus panel. The resulting IC_{50} and IC_{80} values for each antibody were compared to historical data available in the CATNAP database. Only those antibodies exhibiting less than a threefold deviation in IC_{50}/IC_{80} values relative to the reference data were included in subsequent functional analyses.

Eukaryotic cell lines

Policy information about cell lines and Sex and Gender in Research

Cell line source(s)	TZM-bl cells (NIH AIDS Reagent Program), HEK293T (American Type Culture Collection), HEK293-6E cells (National Research Council of Canada). The sex of all cells was female.
Authentication	Cell line were authenticated with STR analysis.
Mycoplasma contamination	Cell line was tested negative for mycoplasma contamination.
Commonly misidentified lines (See ICLAC register)	No commonly misidentified cell lines were used in the study.

Animals and other research organisms

Policy information about studies involving animals; ARRIVE guidelines recommended for reporting animal research, and Sex and Gender in Research

Laboratory animals	NOD.Cg-Rag1tm1momIl2rgtm1Wjl/SzJ (NRG) mice were obtained from The Jackson Laboratory

Laboratory animals	B6.Cg-Fcgrttm1Dcr Prkdcscid Tg(FCGRT)32Dcr/DcrJ (FcRn) mice were obtained from The Jackson Laboratory<br>Humanized NOD-Prkdcscid-IL2rgTm1/Rj (NXG-HIS) were obtained from Janvier Labs.
Wild animals	No wild animals were used for this study
Reporting on sex	Sex was not part of the study design.<br><br>For treatment experiments using NOD.Cg-Rag1tm1momIl2rgtm1Wjl/SzJ (NRG) mice obtained from The Jackson Laboratory:<br>20 male mice, 16 female mice<br><br>For determination of antibody PKs in vivo using B6.Cg-Fcgrttm1Dcr Prkdcscid Tg(FCGRT)32Dcr/DcrJ (FcRn) mice obtained from The Jackson Laboratory:<br>12 female mice (04_A06, 10-1074, 3BNC117)<br>10 female mice (04_A06LS, Sotrovimab)<br><br>For treatment experiments using Humanized NOD-Prkdcscid-IL2rgTm1/Rj (NXG-HIS) were obtained from Janvier Labs.:<br>28 female mice
Field-collected samples	No field samples were collected in this study.
Ethics oversight	All procedures involving mice in this study were sanctioned by the State Agency for Nature, Environmental Protection, and Consumer Protection North Rhine-Westphalia (LANUV).

Note that full information on the approval of the study protocol must also be provided in the manuscript.

Clinical data

Policy information about clinical studies
All manuscripts should comply with the ICMJE guidelines for publication of clinical research and a completed CONSORT checklist must be included with all submissions.

Clinical trial registration	Studies have not been registered
Study protocol	Study protocols will be made available on request from the corresponding author.
Data collection	Data collection was carried out at each site using either local data monitoring systems or the centralized online cohort platform, ClinicalSurveys.net. The collected data were then harmonized and consolidated by staff at the University Hospital Cologne.
Outcomes	Non-interventional study. No outcome parameters were defined.

Plants

Seed stocks	No plants were used in this study
Novel plant genotypes	No plants were used in this study
Authentication	No plants were used in this study

Flow Cytometry

Plots

Confirm that:

- [x] The axis labels state the marker and fluorochrome used (e.g. CD4-FITC).
- [ ] The axis scales are clearly visible. Include numbers along axes only for bottom left plot of group (a 'group' is an analysis of identical markers).
- [x] All plots are contour plots with outliers or pseudocolor plots.
- [x] A numerical value for number of cells or percentage (with statistics) is provided.

Methodology

Sample preparation	PBMCs of study individuals were purified by standard density gradient centrifugation using Histopaque (Sigma Aldrich) and LeucoSep tubes (Greiner Bio-one). Cells were sorted at -150°C in 90% (v/v) FBS (Sigma Aldrich) and 10% (v/v) DMSO (Sigma Aldrich). For single B cell sorts, cells were stained with anti-human CD19-AF700 (BD Bioscience), anti-human IgG-APC (BD Bioscience), DAPI (Thermo Fisher), and the HIV-1 env baits BG505.SOSIP.664-GFP and/or biotinylated YU2gp140 (labeled with Streptavidin-PE) for 30 min on ice. Env-reactive CD19+IgG+DAPI- single cells were sorted into 96-well plates containing 4 µl of lysis buffer.
Instrument	FACSAria Fusion (Becton Dickinson)
Software	BD FACSDIVA, FlowJo10
Cell population abundance	Post-sort fractions were not re-analyzed.
Gating strategy	Gating on lymphocytes, live cells, CD19+ and IgG+ and the GFP+ (BG505.SOSIP.664-GFP) or PE+ (for YU2gp140) for HIV-1 reactive cells.

☒ Tick this box to confirm that a figure exemplifying the gating strategy is provided in the Supplementary Information.
